# Evolution of *Deeper Rooting 1-like* homoeologs in wheat entails the C-terminus mutations as well as gain and loss of auxin response elements

**DOI:** 10.1371/journal.pone.0214145

**Published:** 2019-04-04

**Authors:** Almas Ashraf, Obaid Ur Rehman, Shumaila Muzammil, Jens Léon, Ali Ahmed Naz, Fatima Rasool, Ghulam Muhammad Ali, Yusuf Zafar, Muhammad Ramzan Khan

**Affiliations:** 1 PARC Institute for Advanced Studies in Agriculture, National Institute for Genomics and Advanced Biotechnology (NIGAB), NARC, Islamabad, Pakistan; 2 National Centre for Bioinformatics (NCB), Quaid-i-Azam University, Islamabad, Pakistan; 3 Institute of Crop Science and Resource Conservation (INRES), Department of Crop Genetics and Biotechnology, Rheinische Friedrich-Wilhelms University of Bonn, Bonn, Germany; 4 National Institute for Genomic and Advanced Biotechnology, National Agricultural Research Centre, Park Road, Islamabad, Pakistan; 5 Pakistan Agricultural Research Council, Islamabad, Pakistan; University of Naples Federico II, ITALY

## Abstract

Root growth angle (RGA) in response to gravity controlled by auxin is a pertinent target trait for obtainment of higher yield in cereals. But molecular basis of this root architecture trait remain obscure in wheat and barley. We selected four cultivars two each for wheat and barley to unveil the molecular genetic mechanism of Deeper Rooting 1-like gene which controls RGA in rice leading to higher yield under drought imposition. Morphological analyses revealed a deeper and vertically oriented root growth in “NARC 2009” variety of wheat than “Galaxy” and two other barley cultivars “Scarlet” and “ISR42-8”. Three new homoeologs designated as *TaANDRO1-like*, *TaBNDRO1-like* and *TaDNDRO1-like* corresponding to A, B and D genomes of wheat could be isolated from “NARC 2009”. Due to frameshift and intronization/exonization events the gene structures of these paralogs exhibit variations in size. DRO1-like genes with five distinct domains prevail in diverse plant phyla from mosses to angiosperms but in lower plants their differentiation from LAZY, NGR and TAC1 (root and shoot angle genes) is enigmatic. Instead of IGT as denominator motif of this family, a new C-terminus motif WxxTD in the V-domain is proposed as family specific motif. The EAR-like motif IVLEM at the C-terminus of the TaADRO1-like and TaDDRO1-like that diverged to KLHTLIPNK in TaBDRO1-like and HvDRO1-like is the hallmark of these proteins. Split-YFP and yeast two hybrid assays complemented the interaction of TaDRO1-like with TOPLESS—a repressor of auxin regulated root promoting genes in plants—through IVLEM/KLHTLIPNK motif. Quantitative RT-PCR revealed abundance of *DRO1-like* RNA in root tips and spikelets while transcript signals were barely detectable in shoot and leaf tissues. Interestingly, wheat exhibited stronger expression of *TaBDRO1-like* than barley (*HvDRO1-like*), but *TaBDRO1-like* was the least expressing among three paralogs. The underlying cause of this expression divergence seems to be the presence of AuxRE motif TGTCTC and core TGTC with a coupling AuxRE-like motif ATTTTCTT proximal to the transcriptional start site in *TaBDRO1-like* and *HvDRO1-like* promoters. This is evident from binding of ARF1 to TGTCTC and TGTC motifs of *TaBDRO1-like* as revealed by yeast one-hybrid assay. Thus, evolution of *DRO1-like* wheat homoeologs might incorporate the C-terminus mutations as well as gain and loss of AuxREs and other *cis-*regulatory elements during expression divergence. Since root architecture is an important target trait for wheat crop improvement, therefore DRO1-like genes have potential applications in plant breeding for enhancement of plant productivity by the use of modern genome editing approaches.

## Introduction

Root architecture appears as the most relevant target trait for breeding against nutrient and water scarce conditions [1–4]. It pertains to spatial underground distribution of roots in the soil. It is demonstrated by growth rate, extent of branching, biomass, texture, length, width, steepness, shallowness, orientation and root angle in response to gravity. Unlike tap root system in dicots which comprise primary and lateral roots, the fibrous root system of monocots contains wide mass of equally sized roots contributed by seed borne seminal roots or shoot borne crown roots [5–7]. Root depth is an important feature for plant growth as it allows better access to nutrients such as nitrogen and stored water present in deeper layers of soil thus, leading to higher yield [8–10]. It is generally acknowledged that a deeper, thicker and more branched root system with a high root to shoot ratio can enhance the tolerance of rice to water deficit [11, 12]. In cereal crops, deeper rooting is manifested by the combination of a large root growth angle (RGA) which is the angle between the soil surface and the shallowest primary root, and long seminal and nodal roots [13]. Because of direction control, RGA determines whether a plant develops shallow or deep rooting system. Thus, requirement of an efficient, steeper and vertically oriented root system to exploit water expeditiously is a desirable strategy in major food safety crops such as wheat [14]. But this organ i.e., root remained orphan not only in wheat but also in other crops mainly due to lack of investment in the form of projects, underground nature that makes it inaccessible to direct observations or efficient phenotyping and scarcity of genetic data pertinent to root growth and architecture.

Root development is a complex phenomenon that involves constitutive and adaptive mechanisms of genes as well as close *cis-*regulatory correlations with the shoot parts of the plant [15]. However, due to its extreme importance in breeding programs especially under adverse climate, unveiling of molecular mechanism of root development is inevitable. Genetic determinants of root development in rice, maize, *Arabidopsis* and prunes were identified using complementary direct and reverse genetic approaches such as QTL detection, mutant analysis, transcriptomics and functional genomics. Research on root architecture has brought into light the implication of various factors including key genes, hormones, gene networks and nutrients and hitherto, little progress has been made in connecting RGA, steepness and orientation with yield [7, 16–18]. The first report on influence of auxin on root growth angle of lateral growth was published in *Arabidopsis* [19]. Later on in rice, in addition to *DRO1* involved in RGA, 3 other major QTLs including *DRO2*, *DRO3* and *qSOR1* (*quantitative trait locus for SOIL SURFACE ROOTING 1*)) were also mapped that might control the root architecture system under water deficient conditions for greater rice production [20–24]. Negatively regulated by auxin, DRO1enforses downward bending of roots leading to asymmetric growth. The RGA seems to be controlled by elongation of cells in root tips which may facilitate root in bending downward direction. Due to greater expression of DRO1 gene, RGA increases towards gravity. DRO1 gene enabled the scientists to reduce drought problem by allowing the root to penetrate deeper thereby help in increasing yield even under shortage of water. Guseman et al. [25] found that *DRO1*-related genes are present across diverse plant phyla, and fall within the IGT gene family. The IGT family also includes *NGR* (*NEGATIVE GRAVITROPIC RESPONSE OF ROOTS*), *TAC1* (*TILLER ANGLE CONTROL 1*) and *LAZY1*. Previously it was reported that LAZY1 and its orthologs affect the shoot and root gravitropism in rice, *Arabidopsis*, maize and *Medicago* [26–32]. In *Arabidopsis*, there are 6 LAZY genes which influence tropism, auxin gradient and plant architecture in response to gravity [33]. The IGT family manifests 5 domains (I-V) with IGT as conserved motif in the region II. The AtTAC1 and OsTAC1 lack V region. In line with a potential role in root development, *AtDRO1* and *PpeDRO1* expression is restricted to root [25]. Transgenic *Arabidopsis* and peach exhibited deeper root angles and phenotypes under ectopic expression of DRO1-like while mutation of DRO1-like resulted in the growth of lateral roots in horizontal orientation. An EAR-like motif IVLEI at the C-terminus seems to be involved in generating overexpression phenotypes. This EAR-like is present in many other proteins as well as in IGT family including TAC1 and LAZY1 [25]. The DRO1-like is an important gene with potential to improve yield in wheat under drought regimes. However, its cloning, expression analysis and interaction with other proteins involved in root architecture needs to be unraveled before using it as a marker in the new breeding method i.e. genome editing. Moreover, the contribution of *cis*-regulatory elements in the expression and evolution of DRO1-like genes in wheat and barley has not yet been explored.

Wheat and barley are the most important food safety crops cereal crops adapted to diverse climatic conditions. Modern wheat (*Triticum aestivum* L.) is hexaploid with complex genetics comprising AA, BB and DD genomes contributed by wild relatives. In contrast the diploid genome of barley is closely related to one of the wheat genomes. It will be interesting to study the expression patterns of *DRO1-like* paralogs in wheat and role of *cis*-regulatory elements underlying expression divergence since paralogs evolve under similar evolutionary constraints post duplication/triplication as compared to the orthologs [34]. Therefore the major objective of this study was to unveil expression divergence of *DRO1-like* genes driven by *cis*-regulatory elements during the course of evolution in tritici (here refers to *Triticum aestivum*, *Triticum urartu*, *Aegilops speltoids* and *Aegilops tauschii* only).

This study reiterates that 3 *DRO1-like* homoeologs exist in wheat corresponding to AA, BB and DD genomes. Due to deletions, insertions, frameshifts or intronization/exonization events the genomic locus of *TaBDRO1-like* exhibit different exon/intron structures than other paralogs. All the three wheat homoeologs can interact with TPL with different intensities, probably through IVLEM/KLHTLIPNK motif at the C-terminus. Absence of an AuxRE binding motif TGTCTC and core TGTC as well as a coupling AuxRE-like ATTTTCTT motif proximal to the transcriptional start site (TSS) in *TaADRO1-like*, might be responsible for enhanced expression of this gene in root tissues. Conversely, weaker expression of *TaBDRO1-like* in root tips is attributed to stronger binding of these elements by ARF1 as evident form yeast one hybrid assay.

## Results

### Wheat and barley exhibit variations in root length and angle

**Fig 1:**
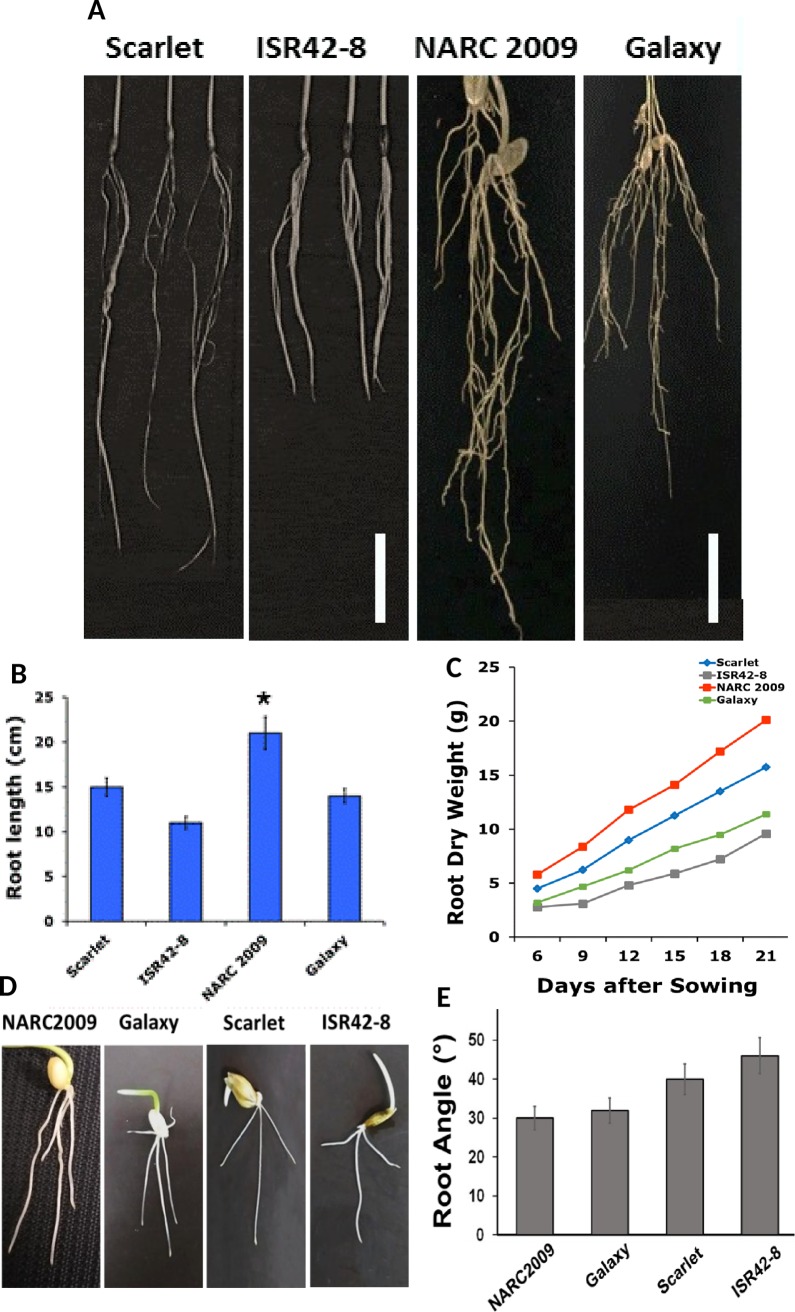
Morphological comparison of root architecture in wheat and barley cultivars. **(A)** Photographs showing the roots of 6-day old plantlets of wheat varieties “NARC 2009 and Galaxy” and barley cultivars “Scarlet and ISR42-8”. Scale bars indicate 4 cm. (**B)** Graph showing root length comparisons of wheat and barley. Error bars indicate the standard deviation. * Significant (P<5%). (**C)** Line graph depicting the trend in total root dry weight in 6 to 21 days old plantlets of wheat and barley. **(D)** Photographs exhibiting root growth angle on MS media in wheat and barley cultivars. **(E)** Graphical representation of variations in root angle of wheat and barley cultivars. Root angle was measured by ImageJ software.

One week old plantlets of two varieties of wheat “NARC 2009 & Galaxy”, and barely cultivars “ISR42-8 & Scarlet” grown in pots were evaluated for root growth architecture (Fig 1A and 1B). Both the species developed fibrous hairy rootlets. NARC 2009 exhibited longer and/or deeper roots than the Galaxy. The average root length was 21cm in NARC 2009 in comparison with 14cm in Galaxy. Barley cultivar Scarlet developed steeper roots with length of 14.9 cm than the wild barley ISR42-8 which produced only 11.4 cm long roots. Interestingly, wild barley generated shortest or the shallowest roots among the selected wheat and barley cultivars while NARC 2009 developed the longest or deepest roots. However, NARC 2009 demonstrated a significance difference at *P* = 0.05. In other varieties, the *P* values remained non-significant. Overall, wheat plants of these varieties demonstrated deeper roots than barley. Moreover, drought tolerant wheat plants were taller with higher survival rate.

Furthermore, we calculated the dry weight of the cultivars from 6 to 21 days at the intervals of 3 days (Fig 1C). A positive correlation was observed between increase in number of days and root biomass in the form of dry weight for all the cultivars. Nevertheless, NARC 2009 revealed the maximum dry weight of 20 g after 21 days, while ISR42-8 showed minimum weight of 9 g. Hence, root dry weight seems to be directly related to root length. An important parameter evaluated was the shape or quantification of root tip angles of 6-day-old plants grown on vertically oriented culture plates (Fig 1D and 1E). In case of wheat, NARC 2009 exhibited narrower root angle and featured more vertically oriented root growth. The average root angle of NARC 2009 was 30° in comparison with 32° in Galaxy. The ISR42-8 showed greater root angle of 48° and promoted less vertically oriented root growth than NARC 2009 and Galaxy.

As *DRO1* gene is involved in RGA in response to gravity under drought conditions in rice, therefore we focused on the isolation and analysis of *DRO1-like* orthologs from wheat and barley.

#### *DRO1-like* orthologs belong to the IGT family of genes in plants

**Fig 2:**
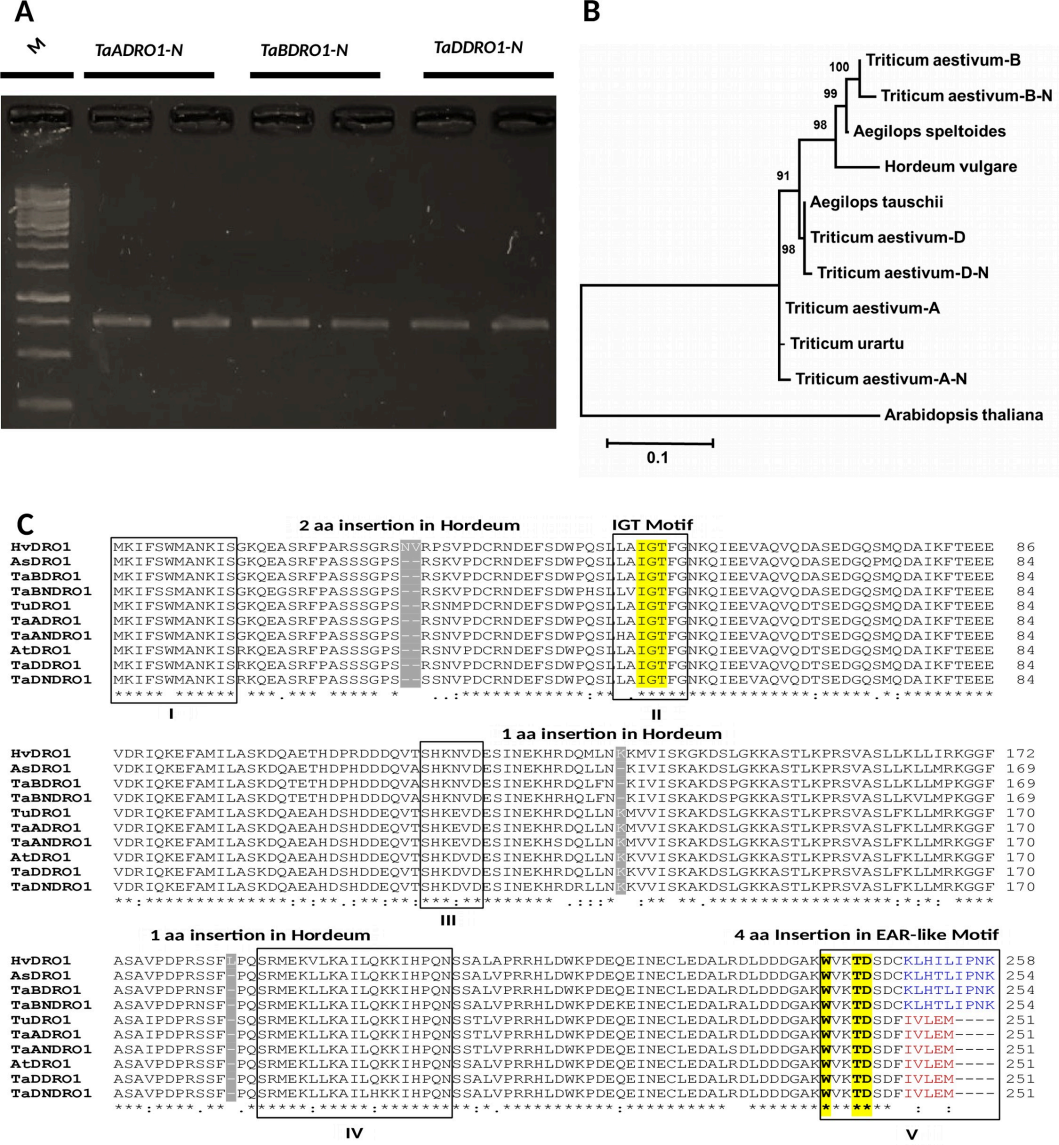
Gene isolation, phylogenetic reconstruction and conservation of DRO1-like homoeologs and orthologs in tritici and close relatives. **(A)** PCR amplification of *TaANDRO1-like*, *TaBNDRO1-like* and *TaDNDRO1-like* from NARC 2009 which is a drought tolerant variety of wheat. M stands for 1kb ladder. (**B)** Phylogenetic tree of *DRO1-like* CDS of Tritici, *Aegilops* and *Hordeum* generated in neighbor-joining algorithm by taking *Arabidopsis* as an outgroup. Bootstrap values for 10000 replicates were calculated for reliability of tree. Scale is given at the base. **(C)** Multiple alignments were generated using ClustalW program in Bio-Edit software. Domains are enclosed in a solid line box and domain number is given under the alignment in each row. One and two amino acid insertions in *Hordeum vulgare* HvDRO1-like are indicated in grey color. Amino acids highlighted in yellow in domain II indicate the IGT motif which is the denominator of this gene family. Bold amino acids in yellow (WxxTD) in the 5^th^ domain at the C-terminus are the newly reported conserved amino acids for distinction of DRO1-like and related proteins. An EAR-like motif comprising 5 amino acids IVLEM is shown in red color but this motif is diverged and extended to 9 aa *KLHTLIPNK* in a few proteins by the addition of IPNK highlighted in blue. This extended motif is specific for AsDRO1-like, TaBDRO1-like, TaBNDRO1-like and HvDRO1-like proteins.

In order to isolate coding sequences of *DRO1-like* homoeologs from selected wheat varieties, genes specific primers corresponding to A, B and D genomes of wheat were employed using root tip cDNA of NARC 2009 as template (S1 Table). PCR amplification following by sequencing and BLAST search in “Ensembl Plants” revealed three *DRO1-like* gene products designated as *TaANDRO1-like*, *TaBNDRO1-like* and *TaDNDRO1-like* (Fig 2A). These newly isolated paralogs clustered with their respective clades comprising *TuDRO1-like* (*Triticum urartu*), *AsDRO1-like* (*Aegilops speltoids*) and *AtaDRO1-like* (*Aegilops tauschii*) in the phylogeny of tritici (Fig 2B). Coding sequences analysis revealed that the size of the newly isolated *DRO1-like* paralogs remained conserved corresponding to their homologs already present in IWGSC database. This size conservation reflects the absence of any deletion, insertion or nonsense mutations in the CDS (S1A–S1C Fig). Pairwise alignment of newly isolated genes showed that the TaANDRO1-like with amino acid length of 251 shared 98.4% identity with TaADRO1-like from database harboring only 4 amino acids substitutions. Similarly, TaBNDRO1-like and TaDNDRO1-like shared 97.8% and 98.6% identity with their counterparts, respectively (S2–S4 Figs).

In order to discern the different structural domains of the DRO1-like proteins, we generated a multiple alignments of the CDS collected from diverse plants including *Triticum*, *Aegilops*, *Hordeum*, cereals, monocots, dicots, *Arabidopsis*, *Amborella*, *Selaginella* and *Physocmitrella*. Fig 2C features 5 distinct domains (I-V) with different types of amino acids for DRO1-like proteins (S5 and S6 Figs). In *Triticum*, *Aegilops* and *Hordeum* all the 5 conserved domains of variable length are observed. However, 3 insertions of total 4 amino acids are prominent in *Hordeum* in the region I, III and IV.

The common denominator of this gene family is an IGT motif [GΦL(A/T)GT] which is present downstream of the N-terminus in the region II. This motif is also conserved in other DRO1 related proteins such as LAZY and TAC1 [25]. A very prominent feature of this alignment is the conservation of 3 amino acid residues WxxTD at the C-terminus in the 5^th^ domain. This motif seems to be more highly conserved in all the DRO1-like proteins than IGT. In *Selaginella*, the IGT is located 28 aa residues further downstream of the conserved region in the 2^nd^ domain. But WxxTD remained intact in 5^th^ domain in all the DRO1-like proteins. Another hallmark of DRO1-like proteins is the occurrence of EAR-like motif (LxLxL) “IVLEM” in the region V at the extreme C-terminus. Ethylene-responsive element binding factor-associated amphiphilic repression (EAR) motif-mediated transcriptional repression is one of the principal mechanisms of plant gene regulation [35]. In DRO1-like genes of dicots, this motif diverged into IVLEI. In tritici however, IVLEM is present in TaADRO1-like and TaDDRO1-like but it is completely altered in TaBDRO1-like and HvDRO1-like with a consensus sequence of LHTLIPNK marked with an addition of IPNK amino acids. The hypothetical progenitor of tritici, *Brachypodium* (in this study) and a primitive plant *Amborella*, kept this IVLEM sequence conserved. Nevertheless, in *Oropetium* grass and other lower plants such as *Physcomitrella* this C-terminal motif is variable in length and sequence composition but it is difficult to distinguish whether these are more DROI-like or LAZY1-like proteins.

Comparison of gene structures of the 17 *DRO1-like* orthologs from various plant phyla indicated the conservation of 5 exons and 4 introns in most of the plants (S7 Fig). But sizes of their genomic loci were variable. Lower plants exhibited larger size of the *DRO1-like* gene due to larger exon and smaller introns. However, a reduction in gene size took place during the course of evolution. For example, *Physcomitrella* encode a protein of 431aa residues followed by *Amborella* of 357 amino acids but rice contains only 221 aa which is even shorter than wheat with 251 residues. It is mainly due to deletions in the coding regions. But in some cases such as *TaBDRO1-like* and *HvDRO1-like* genes of wheat and barley respectively, there might occurred intronization or a merger of exon 4 and 5. Nevertheless, the other two paralogs *TaADRO1-like*, *TaDDRO1-like* and their progenitors (*TuDRO1-like*) (*Triticum urartu*) and (*AtaDRO1-like*) *Aegilops tauschii* kept their exon-intron structures conserved. All the protein coding regions started with ATGAAG exon but *Oropetium* is an exception to this. The first exon of *DRO1-like* gene in *Oropetium* is 576 bp in size containing only two very short introns. The longest 2^nd^ exon of 962bp was detected in *Physcomitrella*. Remarkably, the shortest introns were found in *Oropteium* and *Arabidopsis*. The length variations in gene structures occurred probably due to deletion, insertions and frameshifts or intronization/exonization events.

In spite of length and sequence variations in gene structures, nevertheless, all the DRO1-like proteins kept the IGT signature, EAR-like and WxxTD motifs conserved.

#### DRO1-like interacts with TOPLESS protein

**Fig 3:**
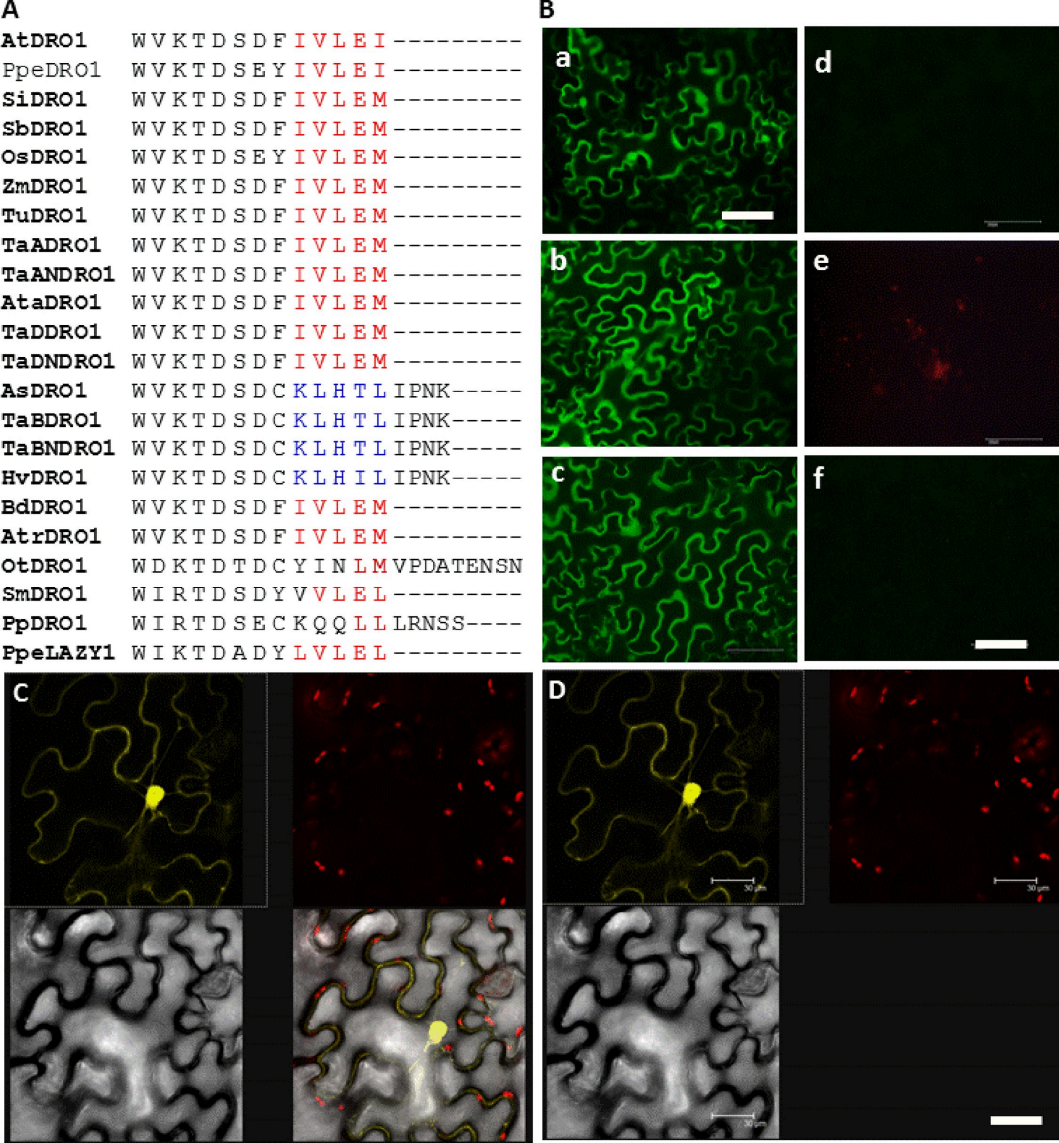
Recruitment of EAR-like motif in interaction of DRO1-like proteins with TOPLESS. **(A)** Amino acid sequence alignment of the 5^th^ domain of the DRO1-like proteins of Tritici, *Aegilops*, *Hordeum*, *Brachypodium*, monocot cereals, *Arabidopsis*, *Prunes*, *Oropetium thomaeum*, *Amborella*, *Selaginella*, *Physcomitrella* and LAZY1 of *Prunus* is shown. The EAR-like motif is highlighted in red letter while blue residues depict an EAR-like motif in AsDRO1-like, TaBDRO1-like and HvDRO1-like with addition of IPNK residues. **(B)** Bimolecular fluorescence complementation analysis. Protein-protein interactions studies of DRO1-like homoeologs with TOPLESS (TPL) protein were carried out in *Nicotiana benthamiana* using Split-YFP analysis. Leaf tissues of *Nicotiana* infiltrated with full-length CDS of TaADRO1-like, TaBDRO1-like and TaDDRO1-like constructs as well as with truncated versions i.e., TaADRO1-like^Δ246^, TaBDRO1-like^Δ245^ and TaDDRO1-like^Δ246^ were scanned for detection of YFP signals using Nikon Digital SIGHT DS-i2, ECLIPSE Ni- fluorescence microscope. The letters a, b and c on the left depict the YFP signals with full length CDS of TaADRO1-like, TaBDRO1-like and TaDDRO1-like, respectively while d, e and f show that YFP signals are vanished with truncated version of DRO1-like proteins. Scale bars represent 100 nm. **(CD)** Confocal Laser Scanning Microscope (CLSM) snapshots of TaBDRO1-like-TPL with white, black and chlorophyll back ground for authenticity of YFP signals.

Previously it was reported that TPL gene involved in lateral branches growth interacts with EAR-like proteins [36]. This EAR-like motif IVLEI at the extreme C-terminus of the DRO1-like proteins in *Arabidopsis* is implicated in narrowing lateral root branching by reducing angle and shortening root lengths as well as in shoot upward curling at leaf margin and alterations in shoot branch angles. With amino acid sequence variations this motif is present in all the DRO1-like and LAZY1-like proteins selected for study (Fig 3A). Interestingly, in TaBDRO1-like and HvDRO1-like proteins of wheat and barley, respectively, this motif is altered and extended as KLHTLLIPNK. It will be very interesting to find whether TaTPL (S8A and S8B Fig) interacts with wheat and barley homoeologs or not. For this purpose, we adopted transient bimolecular fluorescence complementation (BiFC) approach in *Nicotiana benthamiana*. Split-YFP analyses in Fig 3Babc indicate that *Nicotiana* leaves infiltrated with TaADRO1-like, TaBDRO1-like and TaDDRO1-like exhibit YFP signals in cytoplasm, cell membrane and nucleoi, though at different intensities. TaBDRO1-like-TaTPL complementation showed stronger YFP signals than the other two paralogs. As TaBDRO1-like is very similar to HvDRO1-like, therefore these interactions might hold true for HvDRO1-like barley ortholog as well.

In order to test the function of the EAR-like motif we deleted it from all the three wheat DRO1-like proteins. Interestingly, the truncated DRO1-like proteins i.e., TaADRO1-like^Δ246^, TaBDRO1-like^Δ245^ and TaDDRO1-like^Δ246^) were unable to interact with TPL as revealed by complete absence of YFP signals (Fig 3Bdef). Snapshots for the authenticity of YFP signals in black, white and chlorophyll backgrounds using LSCM are given in Fig 3C and 3D. Furthermore, *in vitro* binding assay using Y2H system was also performed to validate the interaction of TaDRO1-like with TPL proteins (S9 Fig).

These results allow us to infer that TaDRO1-like proteins interact with TPL through EAR-like motifs. How this protein i.e. TPL is involved in DRO1-like pathway needs to be empirically validated.

#### *DRO1-like* orthologs exist in diverse phyla of plant kingdom

Since DRO1-like is an important developmental protein recruited in root architecture [22] therefore it may exist in entire plant kingdom. We compiled a dataset of 82 *DRO1-like* orthologs from sequenced genomes of plants representing different phyla. In order to probe the evolutionary relationship among different plants phylogenetic reconstruction was performed in Neighbor Joining algorithm.

A circular tree (S10 Fig) demonstrates the occurrence of *DRO1-like* orthlogs in diverse plants that can be differentiated into 9 to 10 different clades. At the base a small clade of moss *Physcomitrella* and lycophyte *Selaginella* is present. This linage splits into cereals mostly tritici and grasses such as *Oropetium* which harbors a fibrous root system. In the tritici, all the progenitors or genome contributors of modern wheat (*Triticum aestivum*) including *Triticum urartu*, *Aegilops speltoids* and *Aegilops tauschii* are clustered together. Remarkably, barley *HvDRO1-like* is more closely related to *TaBDRO1-like* and *AsDRO1-like*. *Brachypodium* seems to be basal to the tritici. Cereals monocots are next neighbor to palms and banana. Remarkably, Solanaceous plants occupy the position as a sister clade of very diverse plants including *Coffea*, *Populus* and *Jatropha* which in turn are close relatives of lotus and beans instead of *Arabidopsis* and other *Brassica* family members. Cotton is clustered with melons and citrus. Prunes, leguminous crops and other trees show close relationship to each other probably due to their root system architecture similarities. Surprisingly, *Amborella trichopoda* is sister to *Beta vulgaris* though these two have quite different root morphologies.

An important feature that can be observed from this tree is the occurrence of gene/genome duplication of *DRO1-like* orthologs in different plants. In addition to occurrence of genome triplication in modern wheat, this phylogeny reveals genome duplication in *Glycine max*, *Phaseolus vulgaris*, *Brassica napus*, banana, oil palm, grapes and *Nicotiana* etc. The duplicates in certain cases are so diverged that these are not even sister to each other as in the case of *Phaseolus vulgaris*.

This phylogenetic reconstruction reveals wide spread occurrence of *DRO1-like* genes in plants. Their clustering in different clades alludes towards existence of evolutionary relationship in plants with respect to root architecture. Hence expression patterns of these genes are predicted to be divergent even in tritici.

#### Barley and wheat *DRO1-like* paralogs exhibit divergent and stronger expression in root tissues

**Fig 4:**
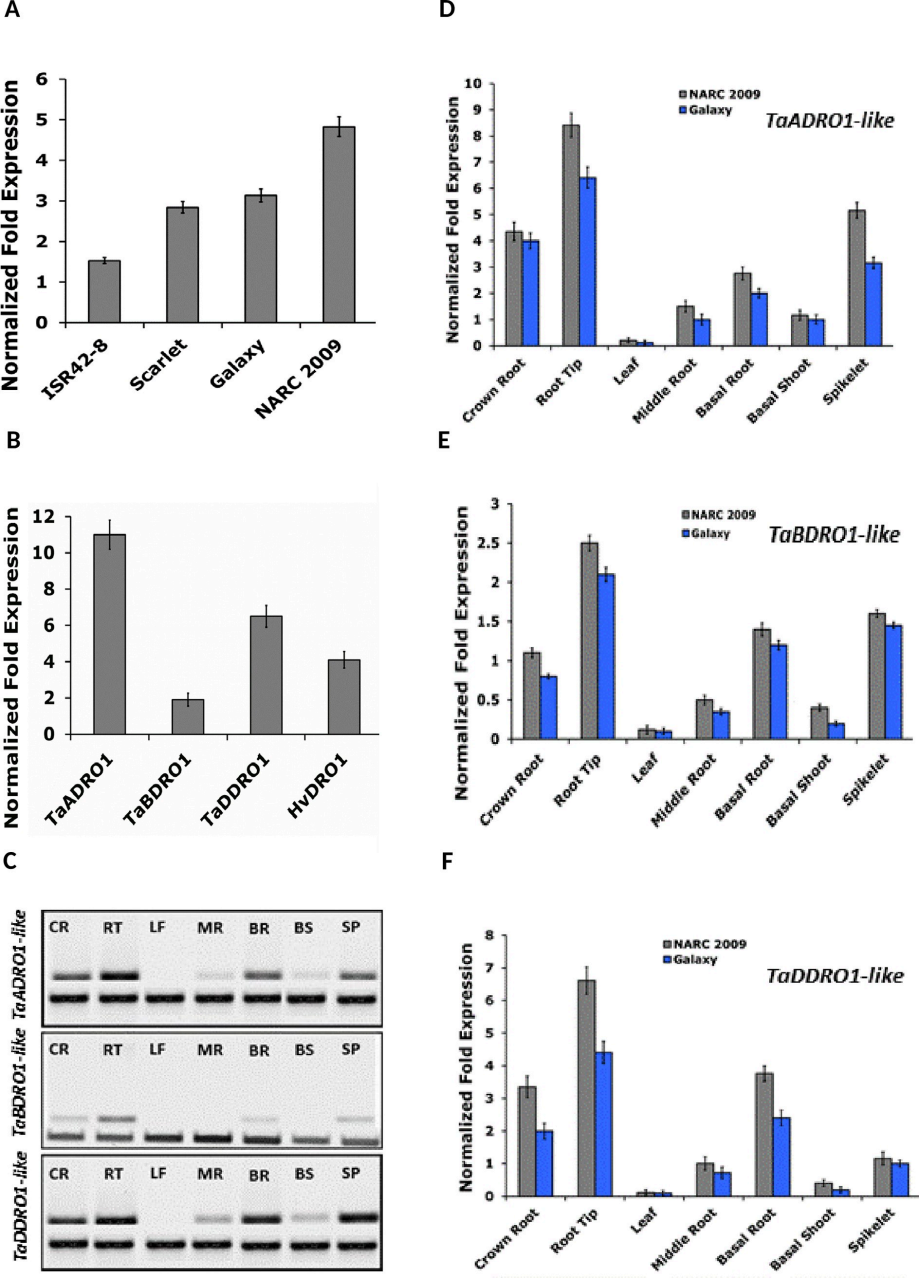
Gene expression patterns of *DRO1-like* transcripts in wheat and barley. **(A)** Quantitative real-time RT-PCR results of the *HvDRO1-like* and *TaBDRO1-like* transcripts in barley and wheat were performed. Normalized fold expression was generated using t *elf* as the internal control. Error bars indicate the standard deviation. (**B)** Comparison of normalized fold expression of four *DRO1-like* orthologs including *TaADRO1-like*, *TaBDRO1-like*, *TaDDRO1-like* of wheat and *HvDRO1-like* of barely. The *tubulin* and *elf* served as endogenous controls for normalized fold expression. (**C)** Semi-quantitative RT-PCR of *DRO1-like* transcripts of *TaADRO1-like*, *TaBDRO1-like* and *TaDDRO1-like* in different tissues of NARC 2009. The *tubulin* served as an internal control. CR, crown root; RT, root tip; LF, leaf; MR, middle root; BR, basal root; BS, basal root; SP, spikelet. (**DEF)** Quantitative real-time RT-PCR of *DRO1-like* transcripts of *TaADRO1-like* **(D)**, *TaBDRO1-like* **(E)** and *TaDDRO1-like* **(F)** in different tissues of NARC 2009 and Galaxy cultivars of wheat. The *tubulin* and *elf* served as endogenous control for normalized fold expression. Color legend is given. Error bars indicate the standard deviation.

Quantitative and semi-quantitative RT-PCR was carried out to compare the expression patterns of *TaBDRO1-like* and *HvDRO1-like* in wheat and barley root tips, respectively. Interestingly, root tips of NARC 2009 and Galaxy showed stronger transcript expression over both the barley cultivars (Figs 4A and S11). Wild barley ISR42-8 displayed the weakest gene expression while NARC 2009 demonstrated the strongest signals. We further compared the expression patterns of *DRO1-like* paralogs of wheat (*TaADRO1-like*, *TaBDRO1-like* and *TaDDRO1-like*) in root tissues of NARC 2009. Surprisingly, the expression of *TaBDRO1-like* was the lowest among three paralogs. On the contrary, *TaADRO1-like* showed the strongest transcript signals in root tips whereas *TaDDRO1-like* featured moderate level of gene expression. Remarkably, the gene expression of *TaBDRO1-like* was even weaker than its close ortholog *HvDRO1-like* in barley.

In order to compare the expression of three paralogs in different tissues includes roots, shoot, leaf and spikelets of NARC 2009 and Galaxy, semi-quantitative and quantitative real time RT-PCR was performed. Fig 4C–4F demonstrates that NARC 2009 surpasses Galaxy in the accumulation of RNA transcripts for the three *DRO1-like* wheat paralogs. All the three genes show stronger expression in root tissues for both the varieties but transcripts are barely detectable in shoot tissues. Root tips exhibit the strongest transcript signals for all three homoeologs in both the varieties. Interestingly, expression of *DRO1-like* wheat paralogs also culminates in spikelets. Nevertheless, all the three paralogs are expressed in root tissue albeit to different level.

These data allow us to infer that besides, showing structural differences, the *DRO1-like* triplicates of wheat also feature divergence in expression. Thus, there is a need to probe into their *cis*-regulatory regions specifically promoters.

#### Gain and loss of cis-regulatory elements in the promoters of *DRO1-like* wheat paralogs might influence the expression divergence

The underlying cause of expression divergence is generally the incremental contribution of *cis*-regulatory elements residing in the promoter and introns [37]. For *cis*-regulatory comparison—using the PipMaker tool—we generated dot plots of -2kb upstream promoter regions of wheat and barley *DRO1-like* orthologs as well as their progenitors including *TuDRO1-like*, *AsDRO1-like* and *AtaDRO1-like* (S12 Fig). All the *DRO1-like* wheat promoters showed divergence from each other and only up to -600bp region near the TSS could be aligned. However, these paralogs exhibited strong conservation with their progenitors, particularly *TaADRO1-like* with *TuDRO1-like* and *TaDDRO1-like* with *AtaDRO1-like*. Interestingly, *TaBDRO1-like* seemed to be quite divergent from its progenitor *AsDRO1-like*. It also shows very less homology with *HvDRO1-like* in comparison with other wheat paralogs although the CDSs of *TaBDRO1-like* and *HvDRO1-like* are quite similar. Interestingly, *HvDRO1-like* promoter is more closely related to *TaDDRO1-like* than *TaBDRO1-like*. This is surprising because their CDS and genes structures are similar, and expression patterns also do overlap. Their expression patterns might be governed by the same *cis*-regulatory motifs. This is one of the speculations that can be put forward for this anomaly.

Previously it was reported that an auxin response factor element (AuxRE) TGTCTC near the TSS in the *DRO1* promoter bound by auxin response factor (ARF1) influences the expression of this gene in rice roots [22]. In this study we mapped the position of TGTCTC and core ARF motif TGTC in the promoter and intronic regions of *DRO1-like* of tritici and other plants (Table 1). The core motif TGTC was located in the intronic and promoter regions more frequently with almost equal rate for all the orthologs. But AuxRE with complete TGTCTC sequence was found only in *Sorghum*, rice, *Amborella* and tritici. Interestingly, among tritici only the *AsDRO1-like*, *TaBDRO1-like* and *HvDRO1-like*, which are sister to each other phylogenetically, contain this motif in the promoter proximal region.

**Table 1:** Identification of AuxREs in *DRO1-like* promoters and introns of Tritici and other plants.

Sr. No.	Gene	TGTCTC Position in Promoter	TGTCTC Position in Introns	TGTC Position in Promoter	TGTC Position in Introns
1	*AtDRO1*	-1287	-	-36, -634, -1196, -1851	+233
2	*SiDRO1*	-	-	-167, -1414, -1427, -1677	-
3	*SbDRO1*	-1486	+77	-769, -823, -1099	+221, +609, +632, +804, +1037, +2164
4	*OsDRO1*	-375	-	-91	+631, +653, +731, +755, +777, +852, +2618
5	*ZmDRO1*	-	-	-309, -501, -607, -770, -994	+308, +1080, +1382, +1586, +2052, +2857, +2888
6	*TuDRO1*	-	-	-772	+72, +452, +838, +1044, +1059, +1147, +1397
7	*TaADRO1*	-	-	-772, -853	+72, +453, +1046, +1061, +1149, +1406
8	*AtaDRO1*	-	-	-372, -1708, -1726, -1827	+510, +676, +852, +886, +901, +977, +1245
9	*TaDDRO1*	-	-	-1708, -1726, -1827	+510, +676, +852, +886, +901, +977, +1245
10	*AsDRO1*	-1802	-	-367, -753, -809, -938, -1785	+513, +827, +848, +882, +897, +1002, +1015
11	*TaBDRO1*	-1251	-	-742, -798, -926, -1738, -1749, -1841	+509, +824, +845, +879, +894, +999
12	*HvDRO1*	-1967, -1977	-	-364, -1330, -1747	+392, +757, +791, +902
13	*BdDRO1*	-	-	-825	+254, +382, +677, +684, +862, +1018, +1268
14	*AtrDRO1*	-1333	-	-818	+1071, +1155, +4031, +4055, +4574
15	*OtDRO1*	-	-	-205, -350, -929, -1281, -1843	-
16	*SmDRO1*	-	-	-107, -388, -587, -1412, -1669	-
17	*PpDRO1*	-	-	-851, -875, -1482, -1591	+36, +355, +1669

The position of TGTCTC and TGTC are shown in the promoters with “-” sign while in the introns are indicated with “+” sign. The only “-” sign indicates the absence of TFBSs in the respective regulatory sequence. The position of nucleotide upstream of ATG is taken as -1 while that of downstream as +1.

**Fig 5:**
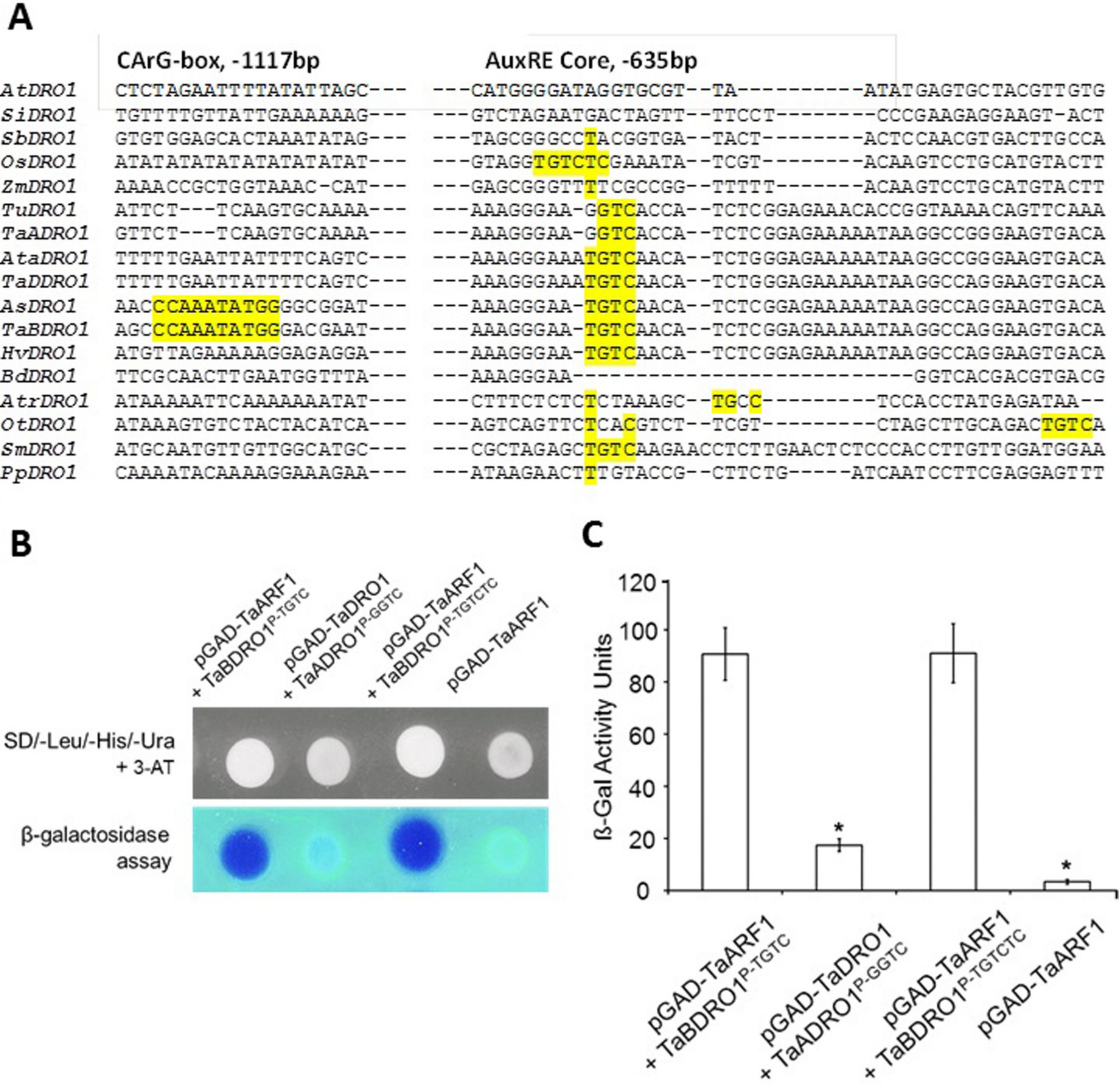
ARF1 binds more strongly with the AuxREs in the promoter region of *TaBDRO1-like*. **(A)** Multiple alignment of partial promoter sequence of *DRO1-like* orthologs from 17 different plants is shown (right panel). The conservation of AuxRE core motif (right panel) and upstream N10–like CArG-boxes (left panel) is depicted. Nucleotides marked as yellow indicate the standard TGTCTC motif and TGTC as core near the TSS while nucleotides underlined as yellow represent the N10-like CArG-box which is a binding site for MADS-box transcription factors. **B)** Yeast 1-hybrid analysis was performed for the detection of binding of ARF1 with *cis*-DNA fragments of *TaDRO1-like* (upper panel). The lower panel indicates non-lethal β-galactosidase assay. **(C)** Calculations of β-galactosidase activity of binding of ARF1 with AuxREs (TGTCTC and TGTC) of *TaDRO1-like*. * Significant (P<5%).

We further probed into the promoters dataset and narrowed down to tritici only. Partial multiple alignment of conserved regions of tritici in Fig 5A (S13 Fig) features a 25bp insertion immediately upstream of the TSS in hypothetical diploid progenitor *Brachypodium*. Interestingly, this insertion encloses an AuxRE core motif TGTC in all the tritici except *TaADRO1-like* and *TuDRO1-like* promoter where it is mutated to GGTC. As *DRO1* is negatively regulated by auxin, therefore presence of TGTCTC upstream and core TGTC in this insertion might be responsible for weaker expression of *TaBDRO1-like* and *HvDRO1-like* genes. On the other hand, their absence in other paralogs *TaADRO1-like* and *TaDDRO1-like* might underlie stronger and moderate expression in root tissues, respectively. Remarkably, further upstream of this AuxRE, a composite AuxRE-like motif is present in all the tritici except TaADRO1-like and TuDRO1-like. The combination of both these motifs in close vicinity is reported to enhance the chances of ARF1 binding. Thus, expression divergence of *TaDRO1-like* paralogs may also be attributed to the possibility of combinatorial interactions of the AuxRE-AuxRE-like motifs. This is very interesting findings because this might hint the relative functional contribution of *DRO1-like* paralogs in roots architecture in wheat. This facilitates in selecting the paralogs that might be used for further functional characterization through genome editing approach.

The binding activity of ARF1 with AuxREs was tested by the Y1H assay using three 200 bp DNA fragments from different regions of *TaDRO1-like* promoters. Fig 5B (upper panel) shows that only yeast clones harboring pGAD-ARF1 plasmid in the presence of *TaBDRO1*^*P-TGTC*^, grow on the medium indicating that ARF1 is indeed able to recognize and interact with AuXREs. In contrast, in the presence of *TaADRO1*^*P-GGTC*^ which contains no AuxRE, yeast growth is undetectable suggesting that this sequence no longer serves as a binding site for ARF1. Remarkably, yeast growth is visible for interaction of *TaBDRO1*^*P-TGTCTC*^ with pGAD-ARF1indicating that true AuxRE has strong binding affinity for ARF1. Non-lethal β-galactosidase assay confirmed the above mentioned interactions (Fig 5C lower panel). The unit activity of β-galactosidase was calculated for the interaction affinity. The results show that there is maximum number of miller units (more than 81) for *TaBDRO1*^*P-TGTCTC*^ with pGAD-ARF1. However, no units of activity were observed for *TaADRO1*^*P-GGTC*^ as this region is devoid of any AuxRE. Moreover, the values obtained were also statistically significant.

Importantly, the TGTC element also occurs at various other positions in the promoter and intronic regions (Table 1). There are 6 TGTCs in the promoter and 7 in the introns (6+7) of *TaBDRO1-like*, and 4+7 in *TaDDRO1-like*. Though *HvDRO1-like* contains 5+5 but it is present singly in highly expressing *TaADRO1-like* and its progenitors *TuDRO1-like*. In *Selaginella*, this motif is present but not existing in *Physcomitrella* (Fig 5A). Its position is a little bit downstream in *Oropetium*. In other cereals and monocots, TGTC core motif is mutated and even absent in *Brachypodium*.

**Fig 6:**
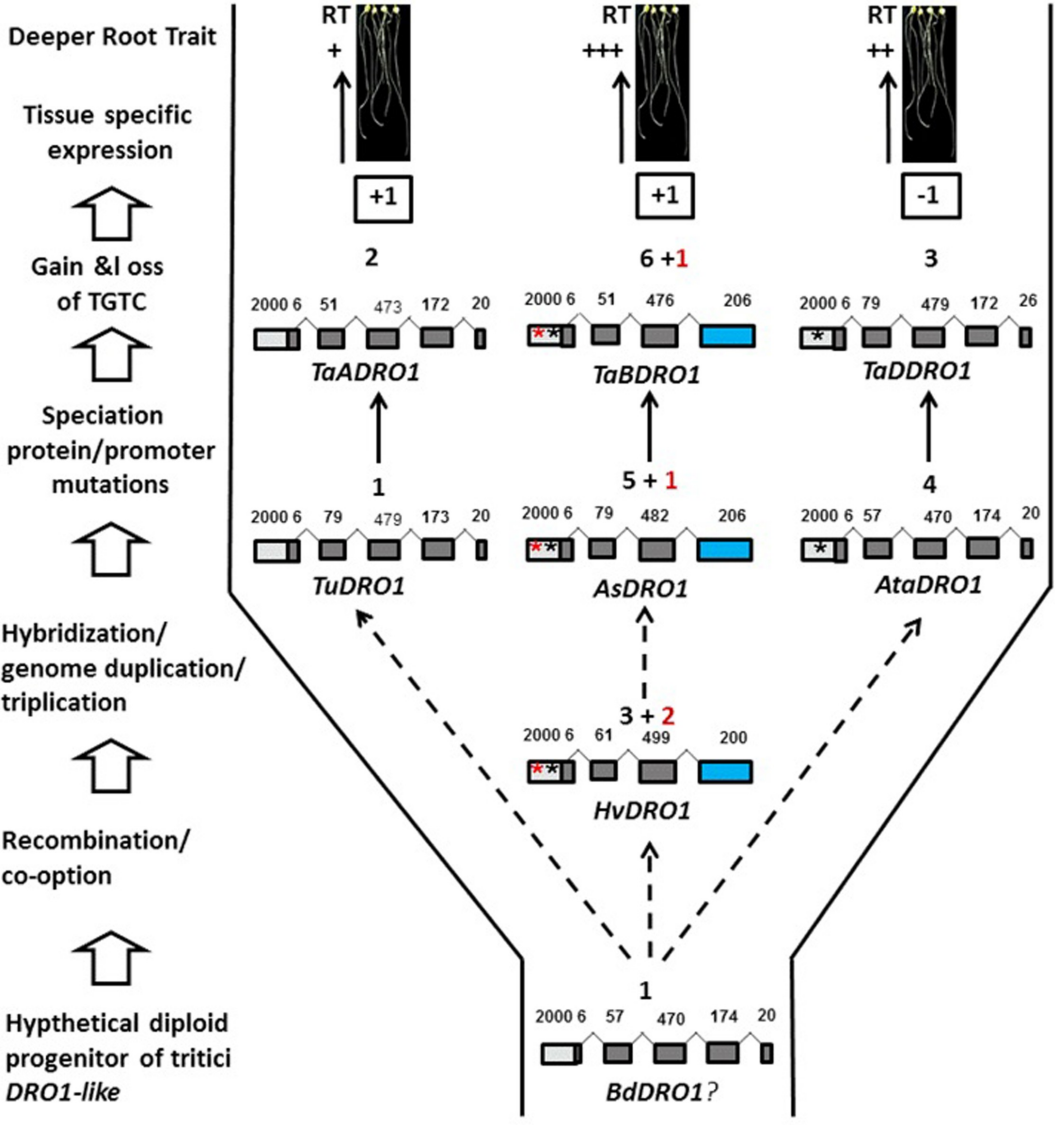
Evolution of *DRO1-like* genes in the Tritici.

Taken together, during the evolution of *DRO1-like* from *TuDRO1-like* to *TaADRO1-like* and *AsDRO1-like* to *TaBDRO1-like* there was a gain of one TGTC in each but in contrast, *TaDDRO1-like* lost one TGTC after hybridization (Fig 6). Thus, *cis-*regulatory regions including promoters and introns diverged, and gain and loss of TGTC might have occurred during genome triplication or hybridization.

A schematic diagram showing the evolution of *DRO1-like* genes in Tritici including *Aegilops* and *Hordeum*. The empty upward arrows at the left side indicate the occurrence of *DRO1-like* evolutionary events from hypothetical diploid progenitor to gene expression stage in the roots of wheat. Dotted arrows symbolize the hypothetical progenitor and originators whereas solid upward arrows mark the changes in gene structures and *cis-*regulatory elements along with variations in gene expression from *TuDRO1-like*, *AsDRO1-like* and *AtaDRO1-like* to *TaADRO1-like*, *TaBDRO1-like* and *TaDDRO1-like*, respectively in wheat paralogs. Exons in gene structures are symbolized by filled boxes while promoters by empty boxes. Upwards arrows between the exon boxes in the gene structures, show the introns, and numbers above indicate the length of exons and promoters in base pairs. Blue boxes depict the reduction in exon number in *AsDRO1-like*, *HvDRO1-like* and *TaBDRO1-like* as well as extension of an EAR-like motif (from IVLEM to KLHTLIPNK) at the C-terminus. Numbers above the nucleotides on gene structures represent the times occurrence of TGTCTC (marked red) and TGTC (marked black) in the promoter regions. An asterisk in the promoter boxes of HvDRO1-like, AsDRO1-like and TaBDRO1-like symbolizes the presence of TGTCTC (red) and TGTC (black) motifs. The TGTC asterisk is also there in *AtaDRO1-like* and *TaDDRO1-like* but not in *TaADRO1-like*, and even in hypothetical progenitor that may be *BdDRO1-like* which is symbolized by a question mark. Gain and loss of TGTCTC and TGTC is indicated by +1 and -1 in the boxes whereas qualitative expression in wheat root tips (RT) is shown as +, ++ and +++ for weaker, moderate and stronger, respectively.

In addition to TGTC, there is loss of MADS-box *cis-*regulatory elements (CArG-boxes) at various positions in the promoters of tritici (S2 Table. These CArG-boxes (CCAAATATGG or CCWWWWWWGG, an N10-like box) might be binding sites for the AGL17 clade member such as AGL21 reported to play a crucial role in lateral roots growth induced by auxin [38]. Different variants of CArG-box were detected in tritici. But interestingly, this element occurred with greater frequency in *TaBDRO1-like* while but remained absent in *TaADRO1-like*. Hence, expression of *TaBDRO1-like* was decreased while that of *TaADRO1-like* increased in the root tips.

We further determined the number and positions of all the plant transcription factor binding sites along the entire length of tritici and barley promoters by taking *BdDRO1-like* as hypothetical progenitor (S3 Table). A total of 597 TFBSs for 35 different types TFs were detected in all the promoters. Remarkably, *AsDRO1-like*, *TaBDRO1-like* and *HvDRO1-like* gained the maximum TFBSs sites while *TaDDRO1-like* the least, when compared with *Brachypodium*. These sites include some of the very important TFs such as MYB84, ABF1, ANT (as highlighted in S12 Fig) ERF2 and NAC69 etc. that play important role in root and shoot development through hormones and stress responsive pathways.

Thus, it is speculated that gain and loss of TFBSs might be responsible for alterations in expression of *DRO1-like* triplicates during root tip development in wheat.

## Materials and methods

### Plant material and growth conditions

Seeds of two varieties of hexaploid wheat (*Triticum aestivum* L.), “NARC 2009” (drought tolerant) and “Galaxy” (drought susceptible) were obtained from Bio-Resources Conservation Institute (BCI) and Crop Sciences Institute, National Agricultural Research Centre (NARC), Islamabad, Pakistan. The Institute of Resource Conservation (INRES) at Bonn University provided the seeds of barley (*Hordeum vulgare* L.) cultivars ISR42-8 (wild barley) and Scarlet (cultivated barley). Seeds were sown in the pots in glasshouses of INRES and National Institute for Genomic and Advanced Biotechnology (NIGAB), NARC, Islamabad. *Nicotiana benthamiana* plants were also grown under standard conditions for split-YFP analysis. The work related to plant morphology and protein-protein interactions was performed at NIGAB but rest of the research work regarding gene isolation, cloning, expression and bioinformatics analyses was executed at INRES as well as in the Genome Editing and Sequencing Lab at National Centre for Bioinformatics, Quaid-i-Azam University, Islamabad, Pakistan.

### Root length, angle and dry weight determination

For determination of root length, 6-days-old germinated seedlings of NARC 2009, Galaxy, ISR42-8 and Scarlet were selected. Plants were shifted from the pots, separated and roots were gently washed with tap water until cleaned. The root length of wheat and barley varieties were measured with a scale and data were recorded as means of replicates.

In order to measure the root angle of selected wheat and barley varieties, surface sterilization of seeds were done with 15% Clorox solution for 12 min. Seeds were rinsed three to four times with sterile distilled water and germinated for 2 d on vertical orientated 0.5 X MS media containing 0.7% agar and 1% sucrose. Seedlings were imaged and root angles were measured using ImageJ software. Furthermore dry weight of the roots was also compared. Seven to 21-days-old plantlets of NARC 2009, Galaxy, ISR42-8 and Scarlet were uprooted at intervals of 3 days. Roots were kept in oven at 37.8°C for 24 hours. They were sealed inside Ziploc bags to avoid regaining of the moisture and weighed on balance after cooling. The procedure was adopted for every single plant separately. Data were recorded as means of replicates. A Student’s T-test was applied to calculate the statistical significance of the bar chart data.

### Genes isolation

Coding sequences of *DRO1-like* genes were retrieved from IPK Barley BLAST Server (http://webblast.ipk-gatersleben.de/barley_ibsc/) and International Wheat Genome Sequencing Consortium (IWGSC; https://www.wheatgenome.org/Tools-and-Resources/Sequences) databases. Primers were designed from the CDS of *TaADRO1-like*, *TaBDRO1-like*, *TaDDRO1-like* and *HvDRO1-like* for gene isolation, expression pattern analysis and protein-protein interaction experiments (S1 Table). Total RNA was extracted from root tips of the NARC 2009, Galaxy, ISR42-8 and Scarlet using TRIZole reagent kit (Thermo Fisher Scientific). The cDNA template was generated by RevertAid kit (Thermo Fisher Scientific). In order to amplify full length coding sequences of *DRO1-like* genes by PCR, rTaq polymerase (TaKaRa, Tokyo, Japan; Code R001) was used. The PCR profile was set at 95°C for 5 minutes, followed by 94°C for 30 seconds, 58°C for 30 seconds and 68°C for 1 minute for 37 cycles. PCR products were resolved and photographed on 1% agarose gel containing ethidium bromide. The purified amplicons were cloned in pTZ57R/T vector by using InsTAclone PCR Cloning Kit (Thermo Fisher Scientific) following the manufacturer’s guidelines, and then sequenced commercially. Similarly, TPL ortholog from wheat was also amplified, cloned and sequenced. Newly isolated TaADRO1-Like-N, TaBDRO1-Like-N and TaDDRO1-Like-N genes have been deposited in NCBI database under accession numbers MK639010, MK639011 and MK639012, respectively,

Genomic loci of 17 *DRO1-like* genes form different plants including tritic, *Aegilops*, *Hordeum* and other higher and lower plants were retrieved using Ensembl genome browser. These sequences were annotated for determination of CDS, UTRs, start-stop codons and intron-exon structures. All the information was documented in the form of a flowchart with nucleotide numbers and positions in base pair unit.

### Bimolecular fluorescence complementation (BiFC)

The TPL protein interacts with other proteins through EAR motif regulated by auxin. As DRO1 is also regulated by auxin, therefore we did the BiFC experiments *in planta* to validate the interaction of TPL with TaADRO1-like, TaBDRO1-like and TaDDRO1-like, and their truncated versions (TaADRO1-like^Δ246^, TaBDRO1-like^Δ245^ and TaDDRO1-like^Δ246^) by removing IVLEM/KLHTLIPNK motif. For this purpose split-YFP system elaborated by Bracha-Drori (2004) and Walter (2004) was employed [39, 40]. The full-length ORFs of these three proteins were first cloned into the pENTR/D-TOPO-201 vector to generate entry clone (Invitrogen) and then Gateway technology (Invitrogen) was used for introducing it into the pBaTL-YFPc and pBaTL-YFPn vectors. The *Agrobacterium tumefaciens* strain GV3101RK was electroporated with the above mentioned 6 constructs. The constructs were co-infiltrated into the 2- to 3-week-old leaves of *Nicotiana benthamiana* from abaxial surface after pinching with a yellow tip. The addition of the p19 viral silencing suppressor was must in the infiltration mixture to avoid co-suppression [41]. The plants with infiltrated leaves were kept under glasshouse conditions for 2 to 3 days. A normal fluorescence microscope Nikon Digital SIGHT DS- i2, ECLIPSE Ni-U was used for scanning YFP signals in leaves after three days of growth. YFP signals were also scanned with CLSM (Leica) and snapshots were taken.

#### Generation of yeast 2-hybrid constructs and protein-protein interaction studies

For detecting TaDRO1-like interactions with TPL protein, AH109 strain with two yeast vectors pGBKT7 (bait) and pGADT7 (prey) were employed (Clontech). Y2H *in vitro* assay was done according to the methods described in the yeast protocols handbook (Clontech). A total of 6 yeast constructs, two each for TaADRO1-like, TaBDRO1-like and TaDDRO1-like, and their truncated versions (TaADRO1-like^Δ246^, TaBDRO1-like^Δ245^ and TaDDRO1-like^Δ246^) were tested. Autoactivation and autobinding were tested by co-transformation TaDRO1-like-TPL proteins with empty bait and prey vectors. Finally, all the constructs were co-transformed into yeast strain AH109 to study their interactions with each other. High-stringency (SD/-Trp-Leu-His-Ade) yeast media were used for plating. The Y2H analysis and interaction matrix was generated as mentioned in Ihsan et al. [42].

### Gene expression

Gene expression patterns of *DRO-like* in wheat and barley were discerned by semi-quantitative and quantitative real-time RT-PCR using real-time PCR machine 7500 Fast (ABI). Total RNA was extracted from different tissues of wheat and barley cultivars using TriZole reagent (Invitrogen). These tissues included crown root, root tip, leaf, middle root, basal root, basal shoot and spikelet. A reaction volume of 25μl was used for each sample. The *elf* and *tubulin* served as endogenous controls. Primers specific to *TaADRO1-like*, *TaBDRO1-like*, *TaDDRO1-like* and *HvDRO1-like* were used on cDNA templates, and RT-PCR was performed as described by Khan et al. [43]. The relative expression of *DRO1-like* transcripts was compared in wheat and barley cultivars. The transcript abundance was also compared for three wheat homoeologs in root tips of NARC 2009. Three biological as well as technical replicates were undertaken. Semi-quantitative RT-PCR products were resolved on 1.5% agarose gel whereas normalized fold expression of real-time PCR products was attained using 7500 Fast Software v2.3.

### Sequence analysis and phylogenetic reconstruction

Newly isolated sequences of *TaANDRO1-like*, *TaBNDRO1-like* and *TaDNDRO1-like* were analyzed using Bio-Edit tool. Sequences were BLAST-searched and only sequences with maximum number of hits with quality score were selected. Complete coding sequences were assembled. In addition to newly isolated sequences, the retrieval of both the coding and promoter sequences of *DRO1-like* orthologs from the different phyla of plant kingdom with sequenced genomes (82 around) including mosses, lycophytes, *Amborella*, *Oropetium* grass, tritici, monocots, dicots, rosids, astrids etc. was carried out using NCBI, Ensembl genome browser [44], (http://www.ensembl.org), IWGSC databases and IPK barley blast server. For sequence analysis, different datasets were generated for titici, monocots, dicots and entire plant phyla. Multiple and pairwise alignments were generated using ClustawlW program in the Bio-Edit Sequence Alignment Editor (http://www.mbio.ncsu.edu). Conserved domains and important motifs were highlighted. In order to infer the evolutionary relationship of *DRO1-like* orthologs, a circular neighbor-joining tree with bootstrap replications of 10000 was reconstructed by MEGA6 [45, 46].

### Promoter analysis and identification of *cis*-regulatory elements

Without a special note we retrieved -2kb upstream sequence as a promoter for all the *DRO1-like* orthologs. Among these, most of the promoter sequences consisted of tritici but also included mosses, liverworts, tuft grass, monocots, dicots and rosids etc. We also selected entire genomic loci of the above mentioned plants comprising all the exon-introns for the identification of *cis*-regulatory elements. Dot plots analyses in PipMaker program (http://pipmaker.bx.psu.edu/pipmaker/) were done [47] for comparison of promoter sequences and identification of regions of close similarity. For this purpose, promoter sequence -2kb upstream of ATG for each gene was inserted in the input box and a dot plot was generated through pairwise alignment. For identification of *cis-*regulatory motifs including auxin response elements (AuxREs), we used Bio-Edit and different online programs such as Mulan (Multiple-sequence local alignment) (http://mulan.dcode.org) with TBA and MultiTF (http://rvista.dcode.org/cgi-bin/mTTP.cgi), and also did it manually [48]. The number and position of TGTCTC and TGTC core motifs was documented for each promoter and intronic region individually. The positions of conserved regions containing AuxREs and AuxRE-like were located and TGTC motif was highlighted.

In order to find all the plant specific TFBSs in the promoter of all the tritici, Mulan program was used. Putative *cis*-acting elements in the individual promoters and downstream regions were predicted using the plant specific *cis*-acting DNA elements in the TRANSFAC (http://www.gene-regulation.com/pub/databases.html) database. The type, number and times occurrence of TFBSs with position was recorded separately for each gene promoter. Presence and absence of TFBSs was compared and gain and loss of TFBBs was inferred from the tables generated.

#### Yeast 1-hybrid assay

In order to confirm the *in vitro* binding of *TaDRO1-like* by *trans*-regulators ARF1 to AuxREs (TGTCTC and TGTC) in the promoters, we performed Y1H analysis. The Y1H assay was carried out using the MATCHMAKER one-hybrid system (Clontech). Three types of *TaDRO1-like* DNA fragments each around 200bp in length were selected. The type 1 fragment contains core AuxRE TGTC of *TaBDRO1-like* while sequence of type 2 fragment was GGTC in case of *TaADRO1* near the TSS. The region towards more proximal promoter from -1100 to -1300 bp containing complete TGTCTC motif was included in the 3^rd^ type of fragment. These three fragments were directly inserted into the multiple cloning sites of reporter plasmids of pLacZi and pHISi-1, respectively. After linearization these three bait constructs were integrated into the genome of yeast strain YM4271. The dual reporter strain was selected and maintained on synthetic dextrose (SD)/-His/-Ura medium. For construction of the pGAD-ARF1 fusion, the cDNA of ARF1 was ligated with GAL4 activation domain in pGAD424 plasmid. Finally constructs were introduced into yeast strain with dual reporter genes, with a blank pGAD424 plasmid as control. After co-transformation, the yeast transformants were tested on SD/-Leu/-His/-Ura medium containing of 3.0 mM 3-amino-1, 2, 4-triazole (3-AT) and 80 mg l^−1^ of 5-bromo-4-chloro-3-indolyl-β-D-galactopyranoside (X-Gal) and 1× BU salt.

Non-lethal β-galactosidase assay was performed as described in Clontech manual. The ORF of ARF1 was fused in-frame with the GAL4 activation domain of the one-hybrid vector pGAD424, and then transferred into yeast cells containing pLacZi-AuxREs plasmids, respectively; the blank pGAD424 plasmid was used as control. The unit of β-galactosidase activity was calculated by the equation of U = 1000×[OD_420_]/(time (in min)×volume (in ml)×[OD_600_]. A Student’s T-test was applied to calculate the statistical significance of the bar chart data.

## Discussion

“*The Power of Movement in Plants*” is famous book in which Charles and Francis Darwin [49] long ago documented the capability of plants for orienting their root growth towards gravity through root tip bending. Gravitropism is necessary for roots to grow into the soil not only for water and nutrient acquisition but to anchor plant as well [50]. Presence of a profuse and deeper root system may have a direct impact on yield under adverse climatic conditions [51]. Therefore, in this study we focused on root system architecture of wheat and barley. When morphologically compared, both the wheat varieties NARC 2009 (drought tolerant) and Galaxy (drought susceptible) showed longer roots in comparison with wild ISR42-8 (wild barley) and Scarlet (cultivated variety). Among all cultivars NARC 2009 demonstrated a deeper root network, more vertical RGA, gain of dry weight, healthier and taller plants.

Development of drought resistant varieties with improved root architecture is the most desirable strategy to increase the crop production [52]. Molecular basis of this orphan trait revealed that DRO1 gene which is negatively regulated by auxin, controls RGA in response to gravity in rice leading to increase in yield under drought imposition [22]. From NARC 2009, we were able to isolate 3 *DRO1-like* paralogs designated as*TaANDRO1-like*, *TaBNDRO1-like* and *TaDNDRO1-like*. Phylogenetically, *HvDRO1-like* of barley is sister to *TaBDRO1-like* of wheat. Comparison of gene structures of the 17 *DRO1-like* orthologs from various plant phyla indicated the conservation of 5 exons and 4 introns in most of the plants. But size of the gene structures remained variable. The length variations in gene structures occurred probably due to deletion, insertions and frameshifts or intronization/exonization events.

Here we found that *DRO1-like* proteins are present across diverse plant phyla and fall within the IGT gene family [26]. LAZY, NGR and TAC1 are also reported as member of this family. These genes control orientations of above and underground plant parts. Six LAZY genes (LZY1-6) with differential expression in various tissues were identified in *Arabidopsis* by Taniguchi et al. [32]. *Arabidopsis* LAZY1 family plays a key role in gravity signaling within statocytes and in branch angle control of roots and shoots. Yoshihara and Spalding [33] identified the role of LAZY family in mediating the effects of gravity on auxin gradients and plant architecture. The previous name of LAZY3 was AtDRO1 or AtNGR2. LAZY and TAC1 contain IGT motif but their function is to control lateral shoot development rather than RGA in monocots and dicots [53–56, 27, 57–59]. In lower plants such as *Physcomitrella* and *Selaginella*, the differentiation between LAZY, TAC1 and DRO1-like proteins remains enigmatic.

Though IGT motif is reported as the signature for this family but our study revealed that another motif WxxTD in the 5^th^ domain can also act as the prominent feature of this family because its position is more conserved across the entire plant phyla than the IGT of 2^nd^ domain. Another common feature of this family is an EAR-like motif at the extreme C-terminus. This motif is present in numerous co-repressors that show co-repression through interaction with TPL protein [60]. Best known for interaction with auxin-regulated proteins such as BODENLOS (BDL) involved in root development, TPL interacts with other transcription factor complexes involved in various other pathways in plants [61–66] thereby converting them to transcriptional repressors [63]. These protein-protein interactions are mediated by a small conserved motif known as the ethylene response factor (ERF)-associated amphiphilic repression (EAR) domain [46] with a consensus sequence (L/F)DLN(L/F)xP, which has also been identified in several other transcription factors with repressive activity [46, 67, 68]. Around 219 candidate proteins belonging to 21 transcription regulator families harbor this EAR-like motif and show protein repression [45].

Gruiseman et al. [25] demonstrated that C-terminus motif IVLEM in DRO1-like is required for lateral root phenotypes in *Arabidopsis*. It also narrows the lateral root growth angles. In the present study, through split-YFP and Y2H analyses we were able to confirm that all the 3 wheat paralogs could interact with TPL protein with different intensities. Remarkably, the difference of IVLEM in TaADRO1-like and TaDDRO1-like, and KLHTLIPNK in TaBDRO1-like did not affect the interactions spectrum of TaDRO1-like with TPL. But TaDRO1-like proteins with removed IVLEM/ KLHTLIPNK abolished the interactions. Thus, IVLEM is recruited in interaction with TPL. Root architecture seems to be trait controlled by a network of genes and this is what our protein-protein interaction studies reveal. How this interaction can change the function in any pathway with repressing activity remained to be unveiled. We may speculate that it might have a role in auxin flux that negatively regulate the expression of *TaDRO1-like* or block its expression in shoots. Hence, this interaction may serve as a link between roots and shoot development.

The role of *cis*-regulatory elements after duplication/triplication of genes during speciation in expression divergence can hardly be exaggerated [69]. Different models have been proposed and genome duplications seems to be the best option to study the gain and loss of *cis-*regulatory elements as genomic paralogs i.e., homoeologs are provided with the same environment than orthologs [34]. *DRO1-like* triplicates of wheat in comparison with barley seems to be ideal for studying the contribution of *cis*-regulatory elements in expression divergence is proposed by this study. The analysis of promoter and intronic regions in wheat indicated that gain and loss of *cis*-regulatory elements might have contributed in the expression divergence of *DRO1-like* paralogs. Presence of an ARF1 binding motif TGTCTC in *TaBDRO1-like* and *HvDRO1-like* upstream of the TSS at positions -1251bp and -1967, -1977, respectively, might be responsible for stronger gene expression in wheat root tips than other two paralogs. Uga et al. [22] observed this TGTCTC motif near the TSS in rice. Through electrophoretic mobility shift assay (EMSA), they validated it as binding site for OsARF1/OsARF23. These auxin-response elements (AuxREs) occur in upstream regions of some early-auxin-response genes, and ARFs bind AuxREs to regulate the transcription of these genes [70]. Within AuxREs, the TGTC motif makes the greatest contribution to ARF binding strength [71]. In promoter analysis, we detected a conserved region near the TSS where TGTC was conserved in *TaBDRO1-like* and *TaDDRO1-like* but mutated to GGTC in *TaDDRO1-like*. This is speculated to be an important element, which by binding with ARF suppresses the expression of *TaBDRO1-like* and *HvDRO1-like*. Mironova et al. [72] suggested that AuxRE with its coupling sequence AuxRE-like (ATTTTCTT) may form a composite element for stronger binding of ARF. Interestingly, this coupling motif is detected in *TaBDRO1-like* but not in *TaADRO1-like* promoters. Remarkably, through Y1H we are able to empirically validate the binding of TaBDRO1-like TGTC and TGTCTC while no binding of GGTC with ARF1 protein was detectable.

Another important motif that can bind MADS-box TFs i.e. CArG-box (CCWWWWWWGG, other variants also do occur) was detected in *TaBDRO1-like* promoter with greater frequency. Root specific AGL 17 clade MADS-box TF such as AGL21 [44] may bind it. These TFs play a crucial role in lateral root development. In *Arabidopsis*, AGL21 was found positively regulating auxin accumulation in lateral root primordia and lateral roots by enhancing local auxin biosynthesis, thus stimulating lateral root initiation and growth. It is speculated that binding of AGL21 to CArG-motif of *TaBDRO1-like* decreases the auxin level and thus decreasing the expression of these genes in roots. Other *cis*-element binding TFs including NAC69, MYB84, ER, and DOF might also play an important role. Most of these sites were lost in the *TaADRO1-like* and *TaDDRO1-like* but gained in *HvDRO1-like*, *AsDRO1-like* and *TaBDRO1-like*.Thus, gain and loss of these *cis*-regulatory elements might have played a major role in expression divergence among *TaDRO1-like* homoeologs during evolution.

Speculations about the evolution of *DRO1-like* in tritici may be many (Fig 6). Earlier in the history, the hypothetical diploid progenitor, for example *Brachypodium* contains a single *DRO1-like* gene harboring an EAR-like IVLEM C-terminus motif but lacking TGTCTC/TGTC core motif in the promoter region. It went through genome triplication/duplication or hybridization event. *TuDRO1-like* of *Triticum urartu*, *AsDRO1-like* of *Aegilops speltoids* and *AtaDRO1-like* of *Aegilops tauschii* originated through genome triplication from *BdDRO1-like* of *Brachypodium*. It is denoted by dotted line. *HvDRO1-like* of *Hordeum vulgare* seems to be a progenitor of *AsDRO1-like* after *Brachypodium*. During speciation procedure, changes in gene structures such as reduction in number of exons through reading frameshifts in *HvDRO1-like* and *AsDRO1-like*, and other promoter mutations where gain and loss of *cis*-regulatory motifs occurred. In the lineage leading to *TuDRO1-like*, the CDS remained unchanged but there was a gain of one core TGTC (AuxRE) motif upstream of the TSS. During the course of evolution from *TuDRO1-like* to *TaADRO1-like* there was gain of one more TGTC in the proximal promoter though it lacked standard TGTCTC which is a complete sequence motif. Probably due to the absence of TGTCTC and TGTC core motif near the TSS this gene transcript exhibited stronger expression in the root tips that make it a good candidate for the functional control of root architecture in wheat. On the other hand, in the lineages leading to *HvDRO1-like* and *AsDRO1-like*, in addition to 4 bp extension in IVLEM motif there was a gain of 2 TGTCTC motifs in the promoter proximal region, and 3 to 5 TGTCs including one core motif in the conserved region near the TSS. However, *AsDRO1-like* lost one TGTCTC but gained two TGTC. The *TaBDRO1-like* re-gained one more TGTC and maintained standard TGTCTC as well. Gain of this TGTCTC/TGTC might be responsible for the weakest expression of *TaBDRO1-like* and of *HvDRO1-like*. The evolution of *TaDDRO-like* is similar to *TaADRO1-like* except it gained 3 core sequences that include TGTC conserved near the TSS. Hence, this gene exhibited moderate expression in root tips. This gain and loss of *cis*-regulatory elements during the evolution might have played a role in the expression divergence of these *DRO1-like* orthologs in tritici.

In nutshell, the variations at the C-terminus as well as gain and loss of AuxREs and other cis-regulatory elements might have played a role in the evolution of DRO1-like wheat paralogs involved in deeper rooting. Since root architecture is an important target trait for wheat crop improvement therefore, isolation, expression and interaction studies of DRO1-like genes in wheat have potential applications in plant breeding for enhancement of plant productivity. It is imperative to study the regulation of stress responses at cellular level in roots, allele replacement for QTL validation and the epigenetic regulation of roots. The unveiling of gene networks and complete pathway and most importantly expression repertories in wheat/crops in root growth and drought avoidance by using genomic/transcriptomic data is inevitable. In this regard, RNA-seq based high throughput sequencing can be a suitable approach. Drought avoidance strategies by exploiting root growth genes through genome editing technique of CRISPR-Cas9 may be very advantageous for designing annual and perennial crops that are productive in moisture-poor soils. CRISPR-Cas9 can be used to disrupt the DRO1-like genes for producing shallower and more profuse wider root systems in some crops for productively enhancement. Ideally, the expression of DRO1-like can be enhanced using CRISPRa manipulation.

## Supporting information

S1 Fig Isolation of *DRO1-like* genes from NARC 2009 cultivar of wheat. Pairwise amino acid sequence alignments of the newly isolated *DRO1-like* paralogs in comparison with those existing in “Ensembl Plants” browser are shown. (A) TaADRO1-like-N with TaADRO1-like. (B) TaBDRO1-like-N with TaBDRO1-like. (C) TaDDRO1-like-N with TaADRO1-like. Conserved nucleotides are shown as asterisks.
(TIF)

S2 Fig Isolation of *TaADRO1-like* gene from NARC 2009 cultivar of wheat. Pairwise nucleotide sequence alignment of newly isolated *TaADRO1-like-N* with *TaADRO1-like* is shown. Conserved nucleotides are shown as asterisks.
(TIF)

S3 Fig Isolation of *TaBDRO1-like* gene from NARC 2009 cultivar of wheat. Pairwise nucleotide sequence alignment of newly isolated *TaBDRO1-like-N* with *TaBDRO1-like* is shown. Conserved nucleotides are shown as asterisks.
(TIF)

S4 Fig Isolation of *TaDDRO1-like* gene from NARC 2009 cultivar of wheat. Pairwise nucleotide sequence alignment of newly isolated *TaDDRO1-like-N* with *TaADRO1-like* is shown. Conserved nucleotides are shown as asterisks.
(TIF)

S5 Fig Multiple partial protein alignment of DRO1-like paralogs and orthologs in tritici and close relatives. Multiple alignments for first 3 domains (I-III) were generated using ClustalW program in Bio-Edit software. Domains are enclosed in a solid line box and domain number is given under the alignment in each row. One and two aa insertions in *Hordeum vulgare HvDRO1-like* are indicated in grey color. Amino acids highlighted in yellow in domain II indicate the IGT motif which acts as the signature of this gene family. Amino acids highlighted in yellow in domain II indicate the IGT motif which is the denominator of this family of genes.
(TIF)

S6 Fig Multiple partial protein alignment of DRO1-like paralogs and orthologs in tritici and close relatives. Multiple partial alignments for last 2 domains (IV, V) were generated using ClustalW program in Bio-Edit software. Domains are enclosed in a solid line box and domain number is given under the alignment in each row. The bold amino acids in yellow (WxxTD) in the 5^th^ domain at the C-terminus are the newly reported conserved amino acids for DRO1-like and related proteins. An EAR-like motif of 5 amino acids (IVLEM) is shown in red color but this motif is diverged and extended to 9 aa KLHTLIPNK in a few proteins by the addition of IPNK highlighted in blue. This extended motif is specific for AsDRO1-like, TaBDRO1-like, TaBNDRO1-like and HvDRO1-like proteins.
(TIF)

S7 Fig Comparison of gene structures of *DRO1-like* orthologs. Gene structures of 17 *DRO1-like* orthologs from Tritici and other plants were analyzed using Ensembl genome browser. The gene structures comprise CDS from ATG to stop codon including introns. Exons in gene structures are symbolized by filled grey boxes while introns by upward lines between the exonic boxes. The numbers above indicate the length of exons in base pairs. The 5’- and 3’- UTRs are shown as empty boxes at the ends. The first two codons of the CDS are shown as ATGAAG in most of the structures except *Oropetium thomaeum*.
(TIF)

S8 Fig Isolation of TOPLESS (TPL) gene form wheat. Pairwise sequence alignments of the newly isolated *TaTPL-N* with existing sequence of *TaTPL* in Ensembl Plants are shown. **(A)** Nucleotide alignment. (**B)** Amino acid alignment. Conserved nucleotides/amino acids are shown as asterisks.
(TIF)

S9 Fig *In vitro* interaction assay of TaDRO1-like proteins with TPL. **(A)** Protein-protein interactions studies of DRO1-like homoeologs with TOPLESS (TPL) proteins were carried out using yeast two hybrid analysis. The binding domain full-length CDS of TaADRO1-like, TaBDRO1-like and TaDDRO1-like constructs as well as with truncated versions (TaADRO1-like^Δ246^, TaBDRO1-like^Δ245^ and TaDDRO1-like^Δ246^) were co-transformed in yeast and grown on stringent dropout media. Note; no growth of yeast colonies in case of truncated DRO1-like proteins. **(B)** Non-lethal β-galactosidase assay.
(TIF)

S10 Fig Phylogenetic reconstruction of *DRO1-like* genes. A circular neighbor-joining tree of 82 *DRO1-like* orthologs from different phyla of plant kingdom was generated in MEGA6. Bootstrap values of 10000 pseudo replicates are indicated at the nodes of the tree. Each clade of the tree is marked with different plant family name and related species.
(TIF)

S11 Fig Gene expression patterns of *DRO1-like* transcripts in wheat and barley. Semi-quantitative RT-PCR results of the *HvDRO1-like* and *TaBDRO1-like* transcripts in wheat and barley. The *elf* is an internal control. M stands for 1kb leader.
(TIF)

S12 Fig Comparison of *DRO1-like* promoters of Tritici, *Aegilops* and *Hordeum*. Graph showing the results of *DRO1-like* promoter analysis of Tritici, *Aegilops* and *Hordeum*. The promoter sequences -2kb upstream of ATG were retrieved and comparison was performed using the PipMaker program (http://pipmaker.bx.psu.edu/pipmaker/).
(TIF)

S13 Fig Promoter comparison and identification of transcription factor binding sites (TFBSs) in *DRO1-like* promoter sequences of Tritici, *Aegilops* and *Brachypodium*. Partial promoter sequences of DRO1-like of Tritici, *Aegilops* and *Brachypodium* were put in Bio-Edit program for generation of multiple alignment through ClustalW tool. Solid boxes enclose the TFBSs for MYB84, AuxRE-like, core Aux-RE in insertion, ABF1 and ANT marked as yellow. A backward arrow indicates the putative position of the transcriptional start sites (TSS). Note the mutation of AuxRE core i.e. TGTC to GGTC in *TuDRO-like* and *TaADRO1-like*, and also in the AuxRE-like motif (ATTTTCTT to TTTTTAGC) as a coupling motif upstream.
(TIF)

S1 Table List of primer sequences used in the study. Primers used for different experiments including cDNA synthesis, gene isolation, expression and protein-protein interactions are shown. The sequence direction is 5’ to 3’.
(TIF)

S2 Table Identification of different types of MADS-domain binding elements i.e. CArG-boxes in the *DRO1-like* promoters of tritici and closely related plants. Different types of CArG-boxes were detected using Mulan program and their positions were marked. The position of these elements was determined by taking first nucleotide upstream of ATG as -1bp. The core sequence motif is mentioned in capital letters.
(TIF)

S3 Table Identification of all the plant specific regulatory elements in the *DRO1-like* promoters of tritici and closely related plants. For identification of TFBSs, -2kb sequence upstream of translational start site was selected as promoter and analyzed using Mulan program. The position and number of times occurrence of different TFBSs were recorded. The “-” sign indicates the absence of a particular site in that promoter sequence. The total number of TFBSs and their occurrence was also documented as highlighted in bold.
(TIF)

S1 File Supporting Information Zip_Archive. Raw data for measurement of root morphology (Fig 1B and 1C), root angle (Fig 1E) and real time gene expression (Fig 4A–4F) analysis.
(ZIP)

## Abbreviations

DRO1: Deeper Rooting1
ARF: Auxin Response Factor
RGA: Root Growth Angle
AuxREs: Auxin Response Elements

## References

[1] Kong X, Zhang M, De Smet I, Ding Z. Designer crops: optimal root system architecture for nutrient acquisition. Trends in Biotechnology. 2014; 32:597–8. 10.1016/j.tibtech.2014.09.008 25450041

[2] Lopez-Arredondo D, Gonz alez-Morales SI, Bello-Bello E, Alejo-Jacuinde G, Herrera Engineering food crops to grow in harsh environments. F1000Res. 2015; 4:651 10.12688/f1000research.6538.1 26380074 PMC4560252

[3] Sun Y Ning T, Liu Z, Pang J, Jiang D, Guo Z, et al The OsSec18 complex interacts with P0(P1-P2)2 to regulate vacuolar morphology in rice endosperm cell. BMC Plant Biology. 2015; 15:55 10.1186/s12870-014-0324-1 25848690 PMC4340293

[4] Li X, Guo Z, Lv Y, Cen X, Ding X, Wu H, et al Genetic control of the root system in rice under normal and drought stress conditions by genome-wide association study. PLoS Genetics. 2017; 17.1006889. 10.1371/journal.pgen.1006889 PMC5521850 28686596

[5] Hochholdinger F, Wen T-J, Zimmermann R, Chimot-Marolle P, da Costa e Silva O, Bruce W, et al The maize (Zea mays L.) roothairless3 gene encodes a putative GPI-anchored, monocot-specific, COBRA-like protein that significantly affects grain yield. The Plant Journal. 2008; 54(5):888–98. 10.1111/j.1365-313X.2008.03459.x 18298667 PMC2440564

[6] Mc Steen P. Auxin and Monocot development. Cold Spring Harbor Perspectives in Biology. 2010; pp. 001479. 10.1101/cshperspect.a001479 PMC2829952 20300208

[7] De Smet I. Lateral root initiation: one step at a time. New Phytologist. 2012; 193:867–873. 22403823 10.1111/j.1469-8137.2011.03996.x

[8] Wasson A, Richards R, Chatrath R, Misra S, Prasad SS, Rebetzke GJ, et al Traits and selection strategies to improve root systems and water uptake in water-limited wheat crops. Journal of Experimental Botany. 2012; 63(9):3485–98. 10.1093/jxb/ers111 22553286

[9] Lynch JP. Root phenes for enhanced soil exploration and phosphorus acquisition: Tools for future crops. Plant Physiology. 2011;156(3):1041–9. 10.1104/pp.111.175414 21610180 PMC3135935

[10] Lynch JP, Wojciechowski T. Opportunities and challenges in the subsoil: Pathways to deeper rooted crops. Journal of Experimental Botany. 2015; 66(8):2199–210. 10.1093/jxb/eru508 25582451 PMC4986715

[11] Fukai S, Cooper M. Development of drought-resistant cultivars using physiomorphological traits in rice. Field Crops Research. 1995; 40:67–86.

[12] Gowda VRP, Henry A, Yamauchi A, Shashidhar HE, Serraj R. Root biology and genetic improvement for drought avoidance in rice. Field Crops Research. 2011; 122:1–13.

[13] Araki H, Morita S, Tatsumi J, Iijima M. Physiomorphological analysis on axile root growth in upland rice. Plant Production Science. 2002; 5:286–93.

[14] Rich SM, Watt M. Soil conditions and cereal root system architecture: review and considerations for linking Darwin and Weaver. Journal of Experimental Botany. 2013; 64:1193–208. 10.1093/jxb/ert043 23505309

[15] Puig J, Pauluzzi G, Guiderdoni E, Gantet P. Regulation of shoot and root development through mutual signaling. Molecular Plant. 2012; 5:974–83. 10.1093/mp/sss047 22628542

[16] Satbhai SB, Ristova D, Busch W. Underground tuning: quantitative regulation of root growth. Journal of Experimental Botany. 2015; 66(4):1099–112. 10.1093/jxb/eru529 25628329

[17] Vermeer JEM, Geldner N. Lateral root initiation in Arabidopsis thaliana: a force awakens. F1000Prime Reports. 2015; 7:32 10.12703/P7-32 25926983 PMC4371239

[18] Wachsman G, Sparks EE, Benfey PN. Genes and networks regulating root anatomy and architecture. New Phytologist. 2015; 208:26–38. 10.1111/nph.13469 25989832

[19] Rosquete MR, von Wangenheim D, Marhavy P, Barbez E, Stelzer EH, Benkova E, et al An auxin transport mechanism restricts positive orthogravitropism in lateral roots. Current Biology. 2013; 23:817–22. 10.1016/j.cub.2013.03.064 23583551

[20] Uga Y, Okuno K, Yano M. Dro1, a major QTL involved in deep rooting of rice under upland field conditions. Journal of Experimental Botany. 2011; 62:2485–2494. 10.1093/jxb/erq429 21212298

[21] Uga Y, Hanzawa E, Nagai S, Sasaki K, Yano M, Sato T. Identification of qSOR1, a major rice QTL involved in soil-surface rooting in paddy fields. Theoretical and Applied Genetics. 2012; 124:75–86 10.1007/s00122-011-1688-3 21894467

[22] Uga Y, Sugimoto K, Ogawa S, Rane J, Ishitani M, Hara N, et al Control of root system architecture by DEEPER ROOTING 1 increases rice yield under drought conditions. Nature Genetics. 2013a; 45:1097–1102 10.1038/ng.2725 23913002

[23] Uga Y, Yamamoto E, Kanno N, Kawai S, Mizubayashi T, Fukuoka S. A major QTL controlling deep rooting on rice chromosome 4. Scientific Rapports. 2013b; 3:3040. 10.1038/srep03040 PMC3807109 24154623

[24] Uga Y, Kitomi Y, Ishikawa S, Yano M. Genetic improvement for root growth angle to enhance crop production. Breeding Science. 2015; 65; 111–119. 10.1270/jsbbs.65.111 26069440 PMC4430504

[25] Guseman JM, Webb L, Srinivasan C, Dardick C. DRO1 influences root system architecture in *Arabidopsis* and *Prunus* species. The Plant Journal. 2017; 89:1093–105. 10.1111/tpj.13470 28029738

[26] Hollender CA, Dardick C. Molecular basis of angiosperm tree architecture. New Phytologist. 2015; 206:541–56. 10.1111/nph.13204 25483362

[27] Yoshihara T, Iino M. Identification of the gravitropism-related rice gene LAZY1 and elucidation of LAZY1-dependent and–independent gravity signaling pathways. Plant Cell Physiology. 2007; 48: 678–688 10.1093/pcp/pcm042 17412736

[28] Godbole R, Takahashi H, Hertel R. The lazy mutation in rice affects a step between statoliths and gravity-induced lateral auxin transport. Plant Biology. 1999; 1: 379–381

[29] Yoshihara T, Spalding EP, Iino M. AtLAZY1 is a signaling component required for gravitropism of the *Arabidopsis thaliana* inflorescence. The Plant Journal. 2013; 74: 267–279 10.1111/tpj.12118 23331961

[30] Sasaki S, Yamamoto KT. Arabidopsis LAZY1 is a peripheral membrane protein of which the carboxy-terminal fragment potentially interacts with microtubules. Plant Biotechnology. 2015; 32: 103–108

[31] Ge L, Chen R. Negative gravitropism in plant roots. Nature Plants. 2016; 17;2(11):16155 10.1038/nplants.2016.155 27748769

[32] Taniguchi M, Furutani M, Nishimura T, Nakamura M, Fushita T, Iijima K, et al The *Arabidopsis* LAZY1 Family Plays a Key Role in Gravity Signaling within Statocytes and in Branch Angle Control of Roots and Shoots. Plant Cell. 2017; 29(8):1984–1999. 10.1105/tpc.16.00575 28765510 PMC5590491

[33] Yoshihara T, Spalding EP. LAZY Genes Mediate the Effects of Gravity on Auxin Gradients and Plant Architecture. Plant Physiology. 2017; 175(2):959–9693 10.1104/pp.17.00942 28821594 PMC5619908

[34] Li P, Wang Y, Qian Q, Fu Z, Wang M, Zeng D, et al LAZY1 controls rice shoot gravitropism through regulating polar auxin transport. Cell Research. 2017; 17:402–10. 10.1038/cr.2007.38 17468779

[35] Kagale S, Links MG, Rozwadowski K. Genome-wide analysis of ethylene-responsive element binding factor-associated amphiphilic repression motif-containing transcriptional regulators in *Arabidopsis*. Plant Physiology. 2010; 152(3): 1109–34. 10.1104/pp.109.151704 20097792 PMC2832246

[36] Ohta M, Matsui K, Hiratsu K, Shinshi H, Ohme-Takagi M. Repression domains of Class II ERF transcriptional repressors share an essential motif for active repression. The Plant Cell. 2001; 13(8):1959–68. 10.1105/TPC.010127 11487705 PMC139139

[37] Wittkopp PJ, Kalay G. Cis-regulatory elements: molecular mechanisms and evolutionary processes underlying divergence. Nature Reviews Genetics. 2012;13:59–69. 10.1038/nrg3095 22143240

[38] Yu LH, Miao ZQ, Qi GF, Wu J, Cai XT, Mao JL, et al MADS-Box transcription factor AGL21 regulates lateral root development and responds to multiple external and physiological signals. Molecular Plant. 2014; 7(11):1653–69. 10.1093/mp/ssu088 25122697 PMC4228986

[39] Bracha-Drori K, Shichrur K, Katz A, Oliva M, Angelovici R, Yalovsky S, et al Detection of protein–protein interactions in plants using bimolecular fluorescence complementation. The Plant Journal. 2004; 40:419–27. 10.1111/j.1365-313X.2004.02206.x 15469499

[40] Walter M, Chaban C, Schütze K, Batistic O, Weckermann K, Nake C, et al Visualization of protein interactions in living plant cells using bimolecular fluorescence complementation. The Plant Journal. 2004; 40:428–38. 10.1111/j.1365-313X.2004.02219.x 15469500

[41] Voinnet O, Rivas S, Mestre P, Baulcombe D. Retracted: An enhanced transient expression system in plants based on suppression of gene silencing by the p19 protein of tomato bushy stunt virus. The Plant Journal. 2003; 33:949–56. 12609035 10.1046/j.1365-313x.2003.01676.x

[42] Ihsan H, Khan MR, Ajmal W, Ali GM. WsMAGO2, a duplicated MAGO NASHI protein with fertility attributes interacts with MPF2-like MADS-box proteins. Planta. 2015; 241:1173–87. 10.1007/s00425-015-2247-y 25630441

[43] Khan MR, Khan I, Ali GM. MPF2-like MADS-box genes affecting SOC1 and MAF1 expression are implicated in flowering time control. Molecular Biotechnology. 2013; 54(1):25–36. 10.1007/s12033-012-9540-9 22539207

[44] Hubbard T, Barker D, Birney E, Cameron G, Chen Y, Clark L, et al The Ensembl genome database project. Nucleic Acids Research. 2002; 1:38–41. 10.1093/nar/30.1.38 PMC99161 11752248

[45] Kumar S, Nei M, Dudley J, Tamura K. MEGA: a biologist-centric software for evolutionary analysis of DNA and protein sequences. Briefings in Bioinformatics. 2008; 9:299–306. 10.1093/bib/bbn017 18417537 PMC2562624

[46] Saitou N, Nei M. The neighbor-joining method: A new method for reconstructing phylogenetic trees. Molecular Biology and Evolution. 1987; 4:406–25. 10.1093/oxfordjournals.molbev.a040454 3447015

[47] Schwartz S, Zhang Z, Frazer KA, Smit A, Riemer C, Bouck J, et al PipMaker-A web server for aligning two genomic DNA sequences. Genome Research. 2000; 10(4):577–86. 10779500 10.1101/gr.10.4.577 PMC310868

[48] Ovcharenko I, Loots GG, Nobrega MA, Hardison RC, Miller W, et al Evolution and functional classification of vertebrate gene deserts. Genome Research. 2005; 15:37–145. 10.1101/gr.3015505 PMC540279 15590943

[49] Darwin C, Darwin F (1880) (John Murray, London).

[50] Tian H, de Smet I, Ding Z. Shaping a root system: Regulating lateral versus primary root growth. Trends in Plant Science. 2014; 19:426–31. 10.1016/j.tplants.2014.01.007 24513255

[51] Comas LH, Becker SR, Cruz VMV, Byrne PF, Dierig DA. Root traits contributing to plant productivity under drought. Frontiers in Plant Science. 2013; 13:4–442. 10.3389/fpls.2013.00442 PMC3817922 24204374

[52] Xiong L, Wang RG, Mao G, Koczan JM. Identification of drought tolerance determinants by genetic analysis of root response to drought stress and abscisic acid. Plant Physiology. 2006; 142(3):1065–74. 10.1104/pp.106.084632 16963523 PMC1630748

[53] Roychoudhry S, Kepinski S. Shoot and root branch growth angle control-the wonderfulness of lateralness. Current Opinion in Plant Biology. 2015; 23:124–31. 10.1016/j.pbi.2014.12.004 25597285

[54] Yu B, Lin Z, Li H, Li X, Li J, Wang Y, et al TAC1, a major quantitative trait locus controlling tiller angle in rice. The Plant Journal. 2007; 52:891–8. 10.1111/j.1365-313X.2007.03284.x 17908158

[55] Ku L, Wei X, Zhang S, Zhang J, Guo S, Chen Y. Cloning and characterization of a putative tac1 ortholog associated with leaf angle in maize (Zea mays). PLoS ONE. 2011; 6:1–7. 10.1371/journal.pone.0020621 PMC3110200 21687735

[56] Dardick C, Callahan A, Horn R, Ruiz KB, Zhebentyayeva T. PpeTAC1 promotes the horizontal growth of branches in peach trees and is a member of a functionally conserved gene family found in diverse plants species. The Plant Journal. 2013; 75:618–30. 10.1111/tpj.12234 23663106

[57] Yoshihara T, Iino M. Identification of the gravitropism-related rice gene LAZY1 and elucidation of LAZY1-dependent and–independent gravity signaling pathways. Plant Cell Physiology. 2007; 48:678–88. 10.1093/pcp/pcm042 17412736

[58] Dong Z, Jiang C, Chen X, Zhang T, Ding L, Song W, et al Maize LAZY1 mediates shoot gravitropism and inflorescence development through regulating auxin transport, auxin signaling, and light response. Plant Physiology. 2013; 163:1306–22. 10.1104/pp.113.227314 24089437 PMC3813652

[59] Yoshihara T, Spaldin EP, Iino M.AtLAZY1 is a signaling component required for gravitropism of the *Arabidopsis thaliana* inflorescence. The Plant Journal. 2013; 74:267–79. 10.1111/tpj.12118 23331961

[60] Kagale S, Rozwadowski K. EAR motif-mediated transcriptional repression in plants: An underlying mechanism for epigenetic regulation of gene expression. Epigenetics. 2011; 6(2):141–6. 10.4161/epi.6.2.13627 20935498 PMC3278782

[61] Kieffer M, Stern Y, Cook H, Clerici E, Maulbetsch C, Laux T, et al Analysis of the transcription factor WUSCHEL and its functional homologue in antirrhinum reveals a potential mechanism for their roles in meristem maintenance. The Plant Cell. 2006; 18(3):560–73. 10.1105/tpc.105.039107 16461579 PMC1383633

[62] Long JA, Ohn C, Smith ZR, Meyerowitz EM. TOPLESS regulates apical embryonic fate in *Arabidopsis*. Science. 2006; 312:1520–3. 10.1126/science.1123841 16763149

[63] Szemenyei H, Hannon M, Long JA. TOPLESS mediates auxin-dependent transcriptional repression during Arabidopsis embryogenesis. Science. 2008; 319(5868):1384–6. 10.1126/science.1151461 18258861

[64] Gallavotti A, Long JA, Stanfield S, Yang X, Jackson D, Vollbrecht E, et al The control of axillary meristem fate in the maize ramosa pathway. Development. 2010; 137(17):2849–56. 10.1242/dev.051748 20699296 PMC2938917

[65] Pauwels L, Barbero GF, Geerinck J, Tilleman S, Grunewald W, Perez AC, et al NINJA connects the co-repressor TOPLESS to jasmonate signalling. Nature. 2010; 464(7289):788–91. 10.1038/nature08854 20360743 PMC2849182

[66] Zhu Y, Qian W, Hua J. Temperature Modulates Plant Defense Responses through NB-LRR Proteins. PLoS Pathogens. 2010; 6(4):e1000844 10.1371/journal.ppat.1000844 20368979 PMC2848567

[67] Hiratsu K, Matsui K, Koyama T, Ohme-Takagi M. Dominant repression of target genes by chimeric repressors that include the EAR motif, a repression domain, in *Arabidopsis*. The Plant Journal. 2004; 34:733–9. 10.1046/j.1365-313x.2003.01759.x 12787253

[68] Hill DP, Smith B, McAndrews-Hill MS, Blake JA. Gene Ontology annotations: what they mean and where they come from. BMC Bioinformatics. 2008; 9 Suppl 5:S2. 10.1186/1471-2105-9-S5-S2 PMC2367625 18460184

[69] Innan H, Kondrashov F. The evolution of gene duplications: classifying and distinguishing between models. Nature Reviews Genetics. 2010; 11:97–108. 10.1038/nrg2689 20051986

[70] Hagen G, Guilfoyle T. Auxin-responsive gene expression: genes, promoters and cis-regulatory factors. Plant Molecular Biology. 2002; 49(3–4):373–85. 12036261

[71] Ulmasov T, Hagen G, Guilfoyle TJ. ARF1, a transcription factor that binds to auxin response elements. Science. 1997; 276(5320):1865–8. 9188533 10.1126/science.276.5320.1865

[72] Mironova VV, Omelyanchuk NA, Wiebe DS, Levitsky VG. Computational analysis of *auxin* responsive elements in the *Arabidopsis thaliana* L. genome. BMC Genomics. 2014; 15pp.S4.</References> 10.1186/1471-2164-15-S12-S4 PMC4331925 25563792

