# Supplementary figures and images for: Evolution of Deeper Rooting 1-like homoeologs in wheat entails the C-terminus mutations as well as gain and loss of auxin response elements
Source: PLoS One. 2019 Apr 4;14(4):e0214145. doi: 10.1371/journal.pone.0214145 (PMC6448822; doi:10.1371/journal.pone.0214145)

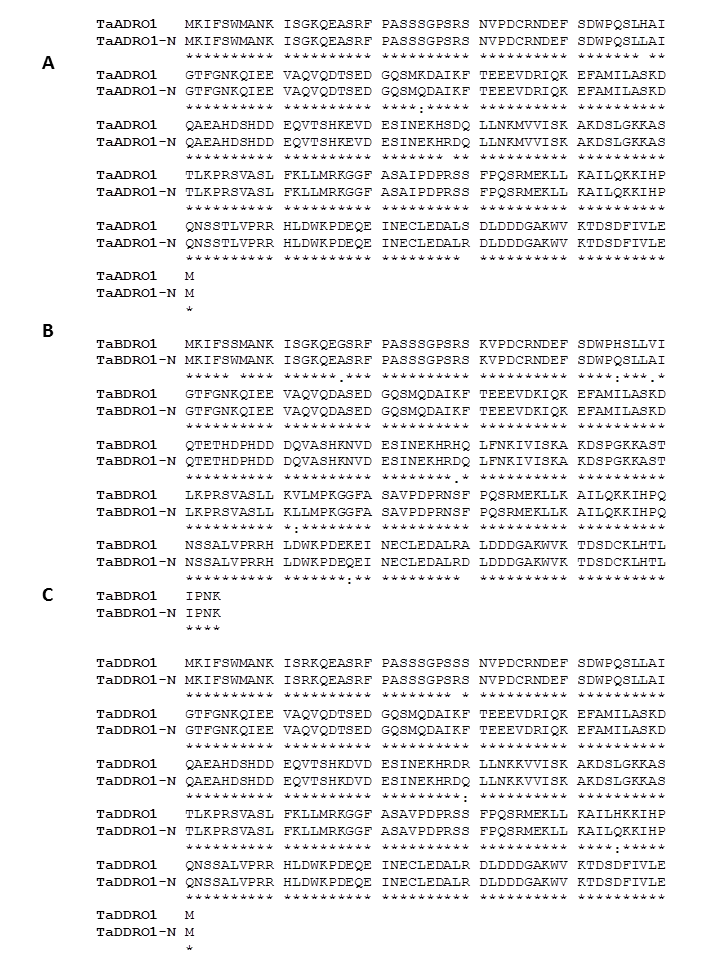

Supplement: S1 Fig — Pairwise amino acid sequence alignments of the newly isolated DRO1-like paralogs in comparison with those existing in “Ensembl Plants” browser are shown. (A) TaADRO1-like-N with TaADRO1-like. (B) TaBDRO1-like-N with TaBDRO1-like. (C) TaDDRO1-like-N with TaADRO1-like. Conserved nucleotides are shown as asterisks. (TIF) [file pone.0214145.s001.TIF]

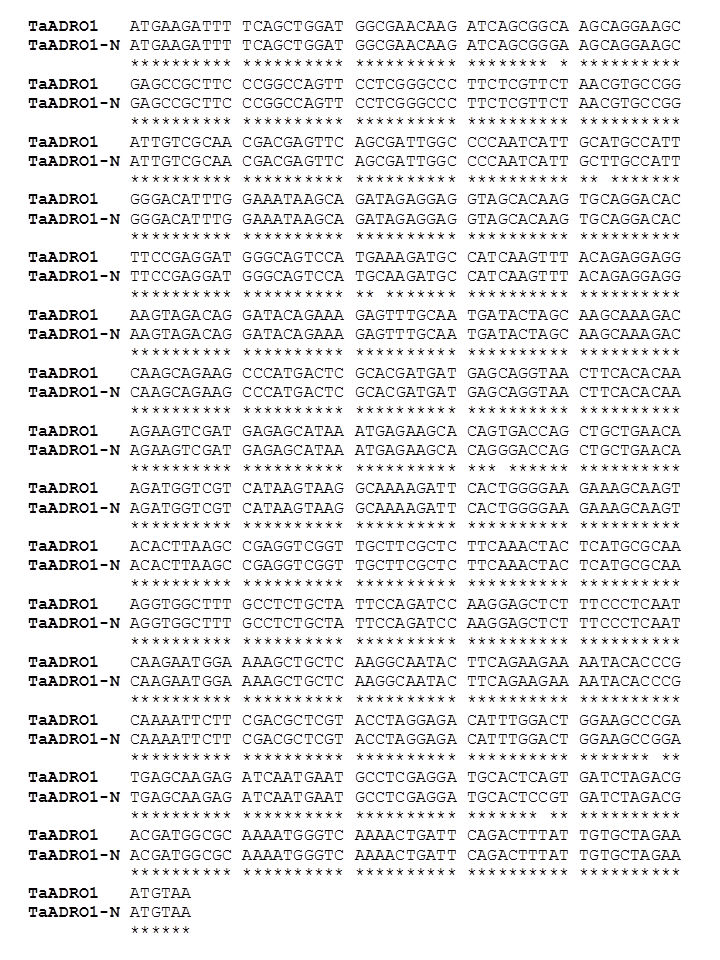

Supplement: S2 Fig — Pairwise nucleotide sequence alignment of newly isolated TaADRO1-like-N with TaADRO1-like is shown. Conserved nucleotides are shown as asterisks. (TIF) [file pone.0214145.s002.TIF]

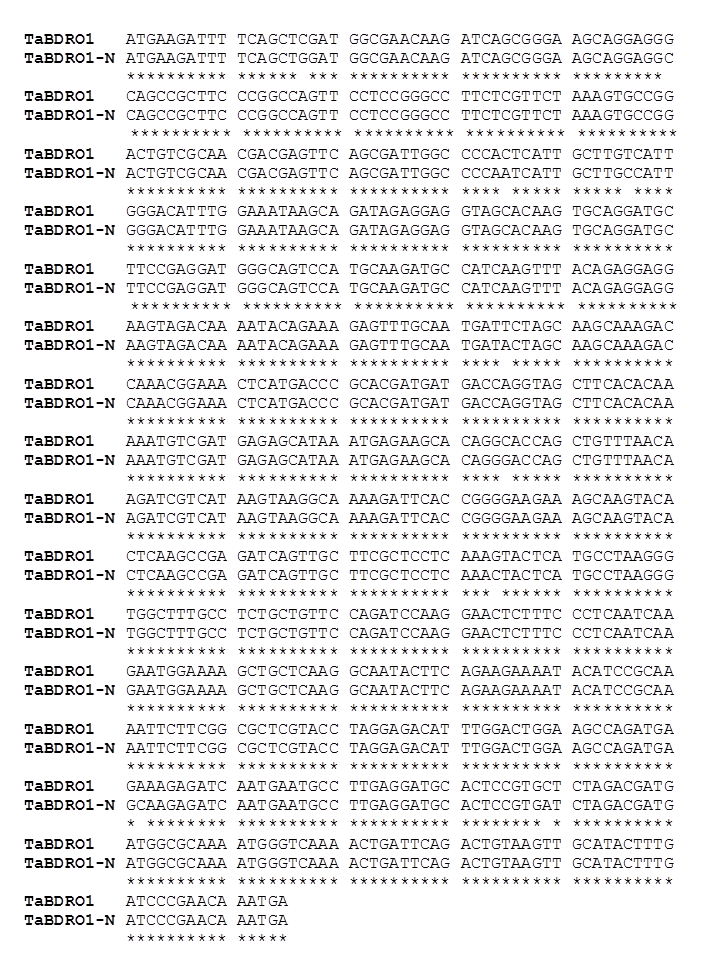

Supplement: S3 Fig — Pairwise nucleotide sequence alignment of newly isolated TaBDRO1-like-N with TaBDRO1-like is shown. Conserved nucleotides are shown as asterisks. (TIF) [file pone.0214145.s003.TIF]

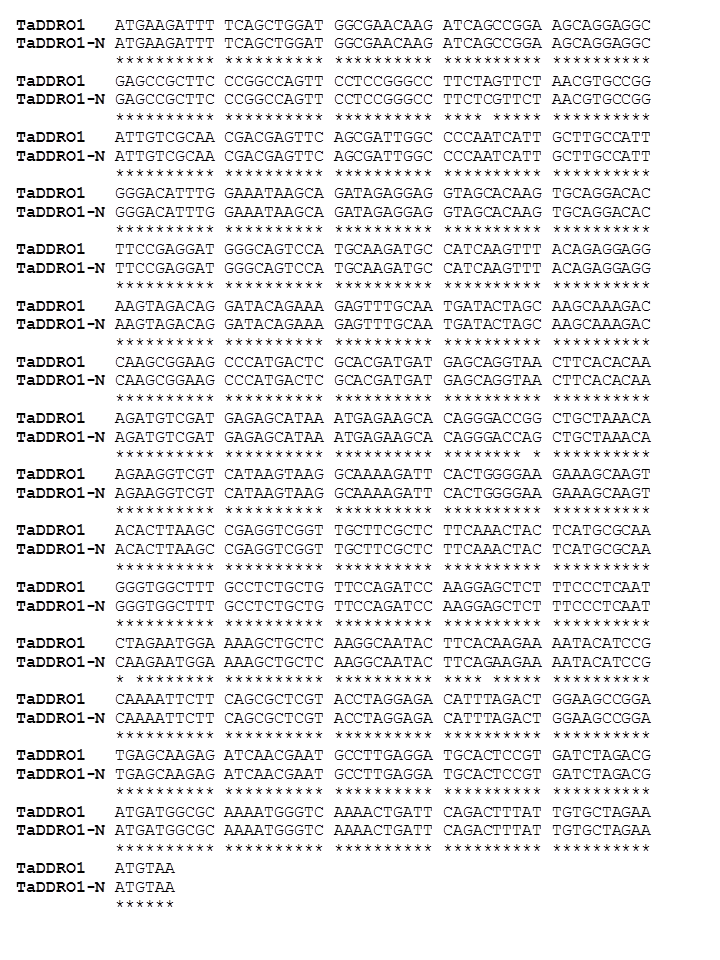

Supplement: S4 Fig — Pairwise nucleotide sequence alignment of newly isolated TaDDRO1-like-N with TaADRO1-like is shown. Conserved nucleotides are shown as asterisks. (TIF) [file pone.0214145.s004.TIF]

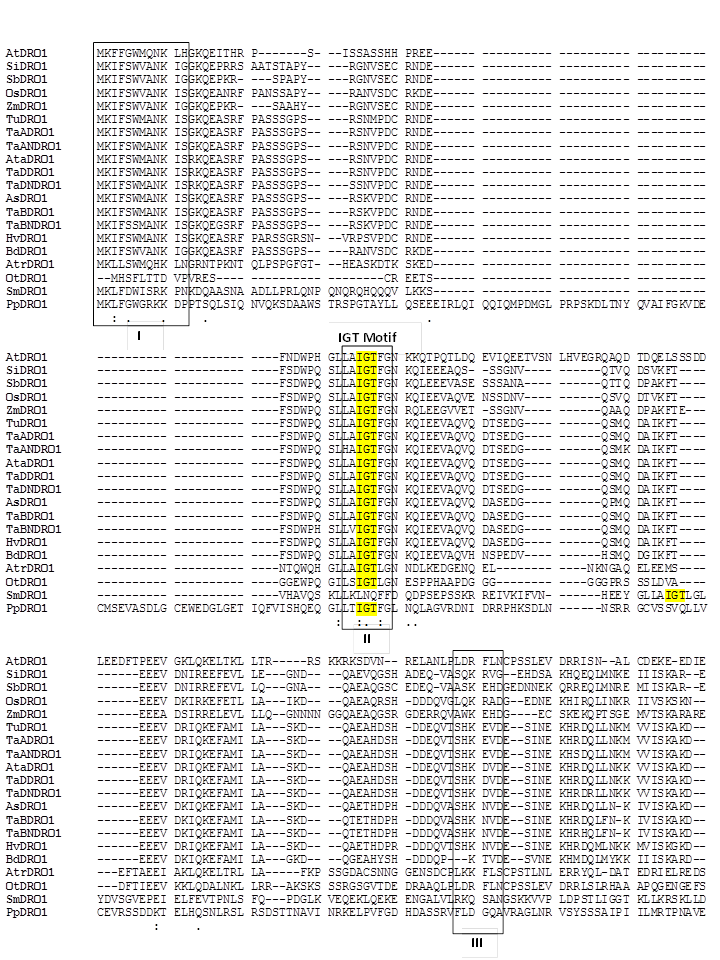

Supplement: S5 Fig — Multiple alignments for first 3 domains (I-III) were generated using ClustalW program in Bio-Edit software. Domains are enclosed in a solid line box and domain number is given under the alignment in each row. One and two aa insertions in Hordeum vulgare HvDRO1-like are indicated in grey color. Amino acids highlighted in yellow in domain II indicate the IGT motif which acts as the signature of this gene family. Amino acids highlighted in yellow in domain II indicate the IGT motif which is the denominator of this family of genes. (TIF) [file pone.0214145.s005.TIF]

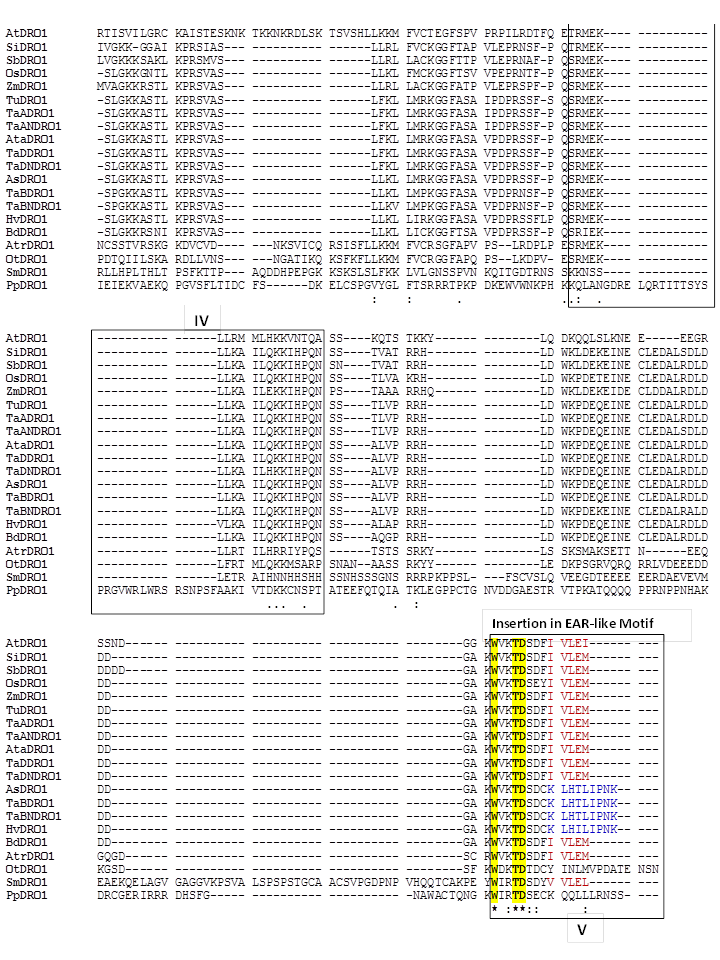

Supplement: S6 Fig — Multiple partial alignments for last 2 domains (IV, V) were generated using ClustalW program in Bio-Edit software. Domains are enclosed in a solid line box and domain number is given under the alignment in each row. The bold amino acids in yellow (WxxTD) in the 5th domain at the C-terminus are the newly reported conserved amino acids for DRO1-like and related proteins. An EAR-like motif of 5 amino acids (IVLEM) is shown in red color but this motif is diverged and extended to 9 aa KLHTLIPNK in a few proteins by the addition of IPNK highlighted in blue. This extended motif is specific for AsDRO1-like, TaBDRO1-like, TaBNDRO1-like and HvDRO1-like proteins. (TIF) [file pone.0214145.s006.TIF]

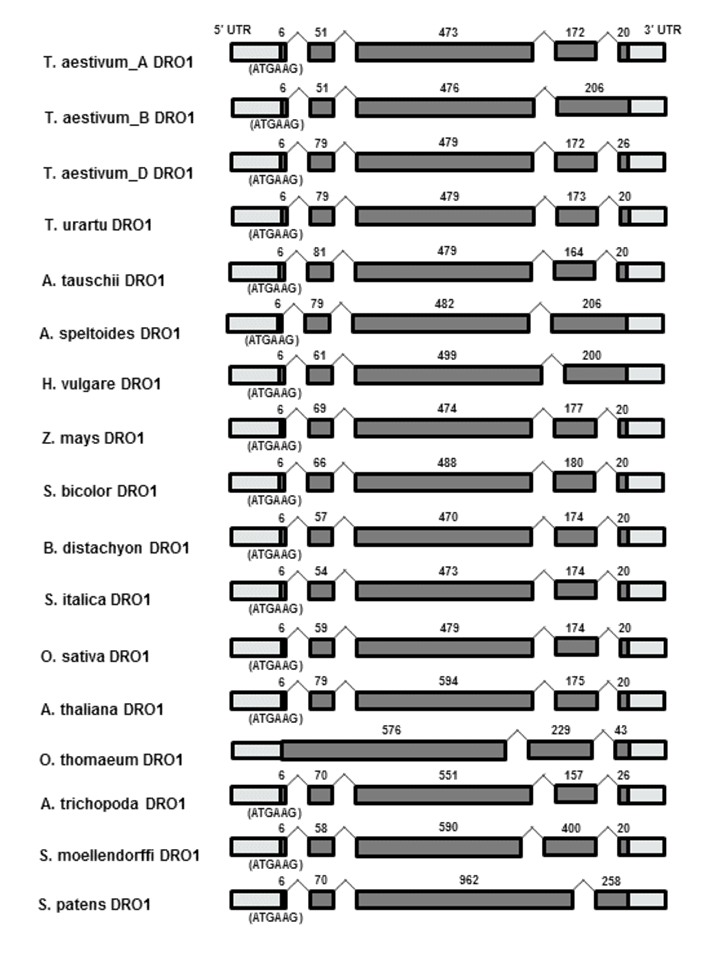

Supplement: S7 Fig — Gene structures of 17 DRO1-like orthologs from Tritici and other plants were analyzed using Ensembl genome browser. The gene structures comprise CDS from ATG to stop codon including introns. Exons in gene structures are symbolized by filled grey boxes while introns by upward lines between the exonic boxes. The numbers above indicate the length of exons in base pairs. The 5’- and 3’- UTRs are shown as empty boxes at the ends. The first two codons of the CDS are shown as ATGAAG in most of the structures except Oropetium thomaeum. (TIF) [file pone.0214145.s007.TIF]

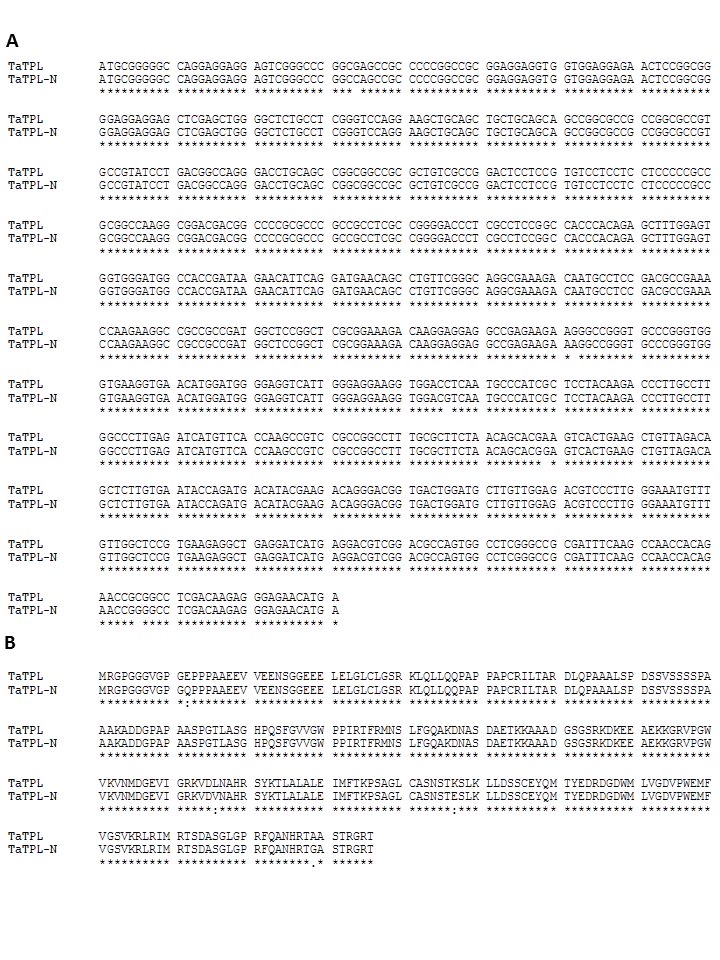

Supplement: S8 Fig — Pairwise sequence alignments of the newly isolated TaTPL-N with existing sequence of TaTPL in Ensembl Plants are shown. (A) Nucleotide alignment. (B) Amino acid alignment. Conserved nucleotides/amino acids are shown as asterisks. (TIF) [file pone.0214145.s008.TIF]

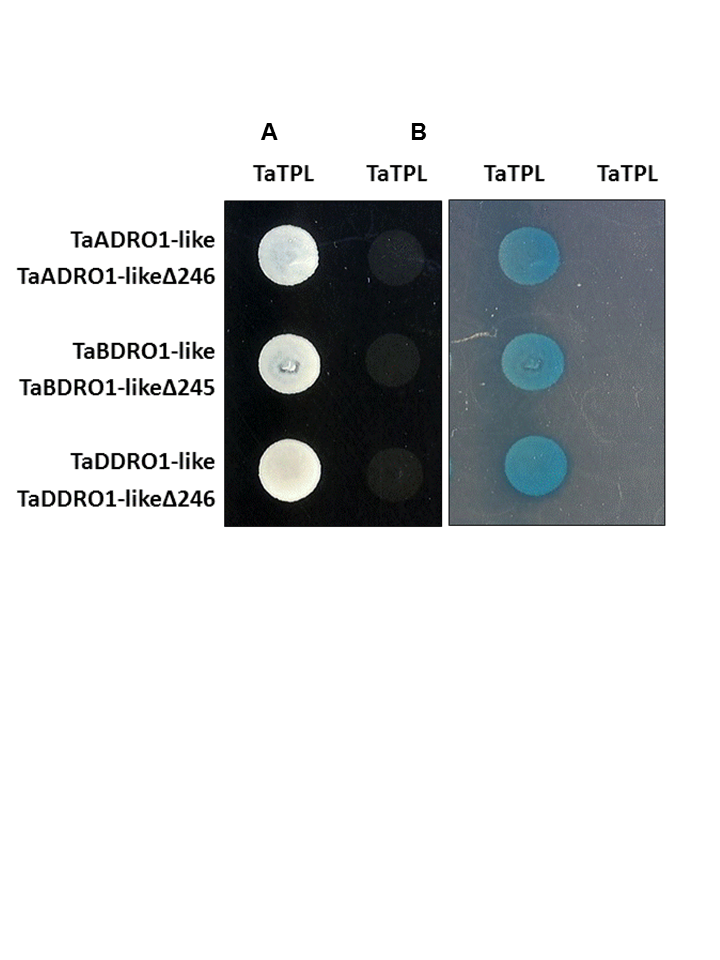

Supplement: S9 Fig — (A) Protein-protein interactions studies of DRO1-like homoeologs with TOPLESS (TPL) proteins were carried out using yeast two hybrid analysis. The binding domain full-length CDS of TaADRO1-like, TaBDRO1-like and TaDDRO1-like constructs as well as with truncated versions (TaADRO1-likeΔ246, TaBDRO1-likeΔ245 and TaDDRO1-likeΔ246) were co-transformed in yeast and grown on stringent dropout media. Note; no growth of yeast colonies in case of truncated DRO1-like proteins. (B) Non-lethal β-galactosidase assay. (TIF) [file pone.0214145.s009.TIF]

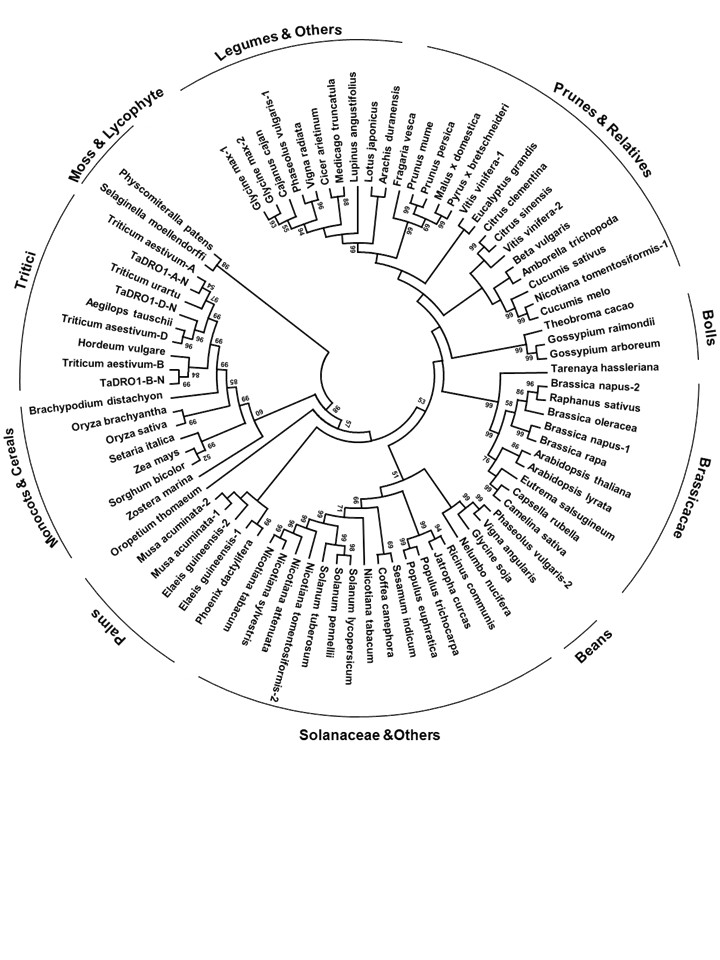

Supplement: S10 Fig — A circular neighbor-joining tree of 82 DRO1-like orthologs from different phyla of plant kingdom was generated in MEGA6. Bootstrap values of 10000 pseudo replicates are indicated at the nodes of the tree. Each clade of the tree is marked with different plant family name and related species. (TIF) [file pone.0214145.s010.TIF]

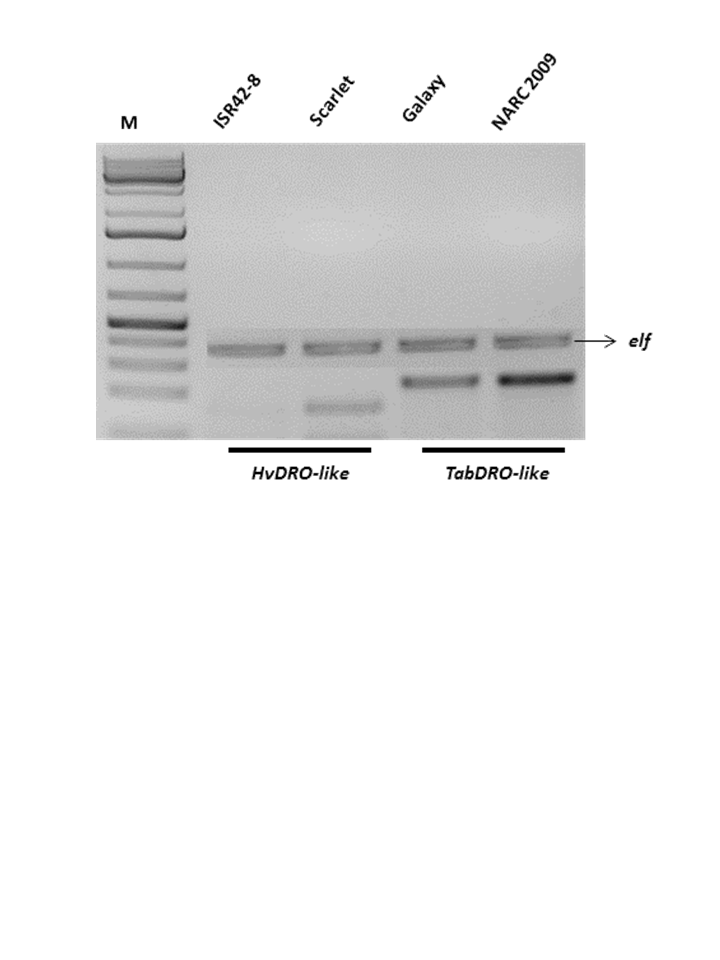

Supplement: S11 Fig — Semi-quantitative RT-PCR results of the HvDRO1-like and TaBDRO1-like transcripts in wheat and barley. The elf is an internal control. M stands for 1kb leader. (TIF) [file pone.0214145.s011.TIF]

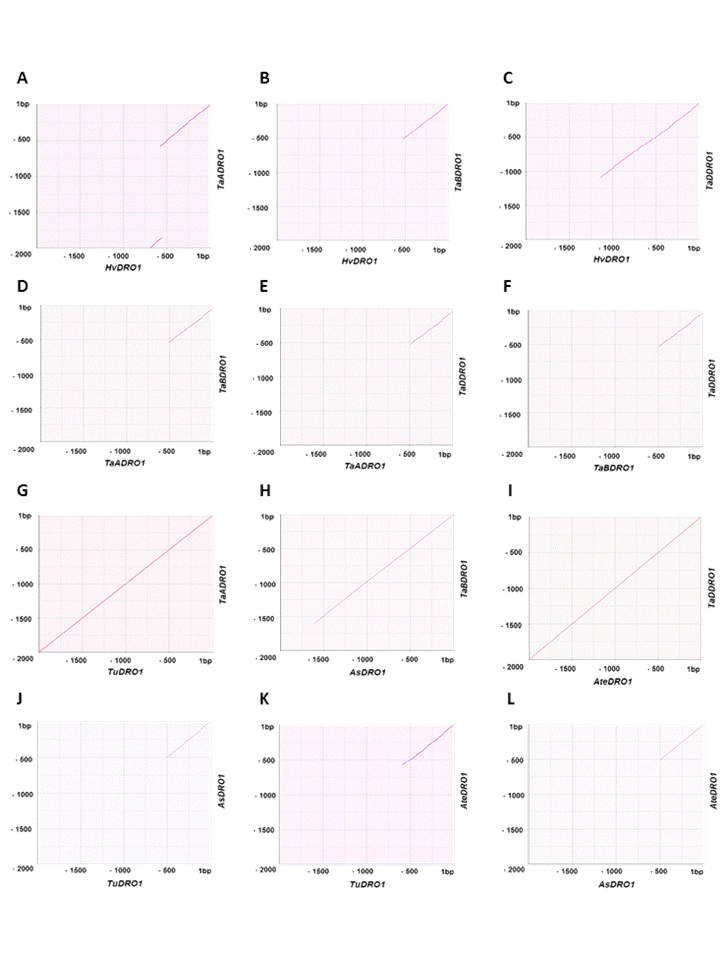

Supplement: S12 Fig — Graph showing the results of DRO1-like promoter analysis of Tritici, Aegilops and Hordeum. The promoter sequences -2kb upstream of ATG were retrieved and comparison was performed using the PipMaker program (http://pipmaker.bx.psu.edu/pipmaker/). (TIF) [file pone.0214145.s012.TIF]

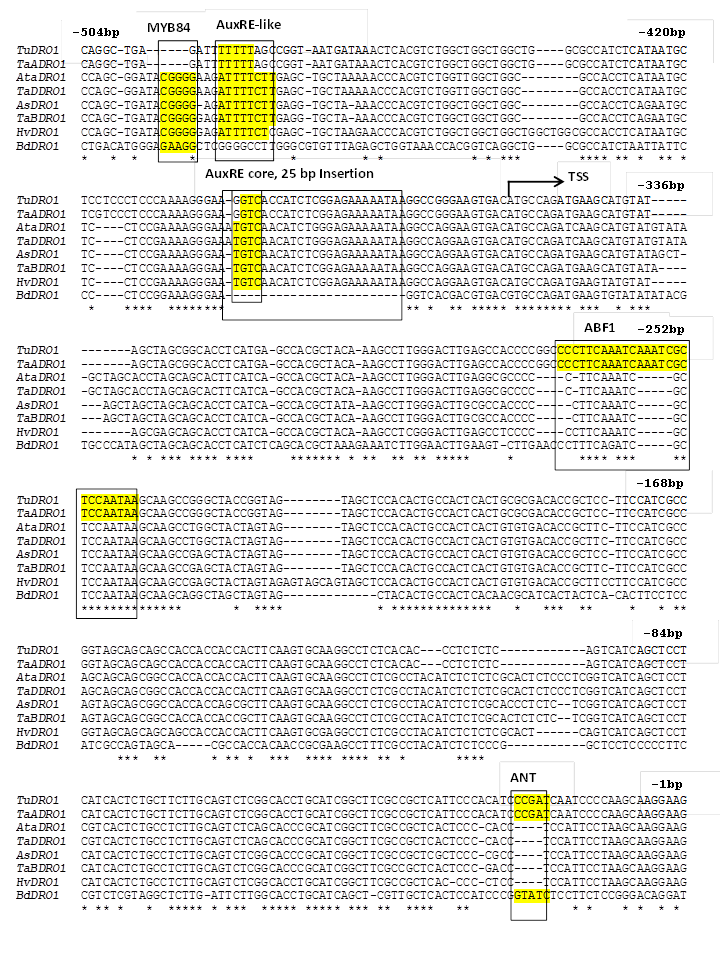

Supplement: S13 Fig — Partial promoter sequences of DRO1-like of Tritici, Aegilops and Brachypodium were put in Bio-Edit program for generation of multiple alignment through ClustalW tool. Solid boxes enclose the TFBSs for MYB84, AuxRE-like, core Aux-RE in insertion, ABF1 and ANT marked as yellow. A backward arrow indicates the putative position of the transcriptional start sites (TSS). Note the mutation of AuxRE core i.e. TGTC to GGTC in TuDRO-like and TaADRO1-like, and also in the AuxRE-like motif (ATTTTCTT to TTTTTAGC) as a coupling motif upstream. (TIF) [file pone.0214145.s013.TIF]

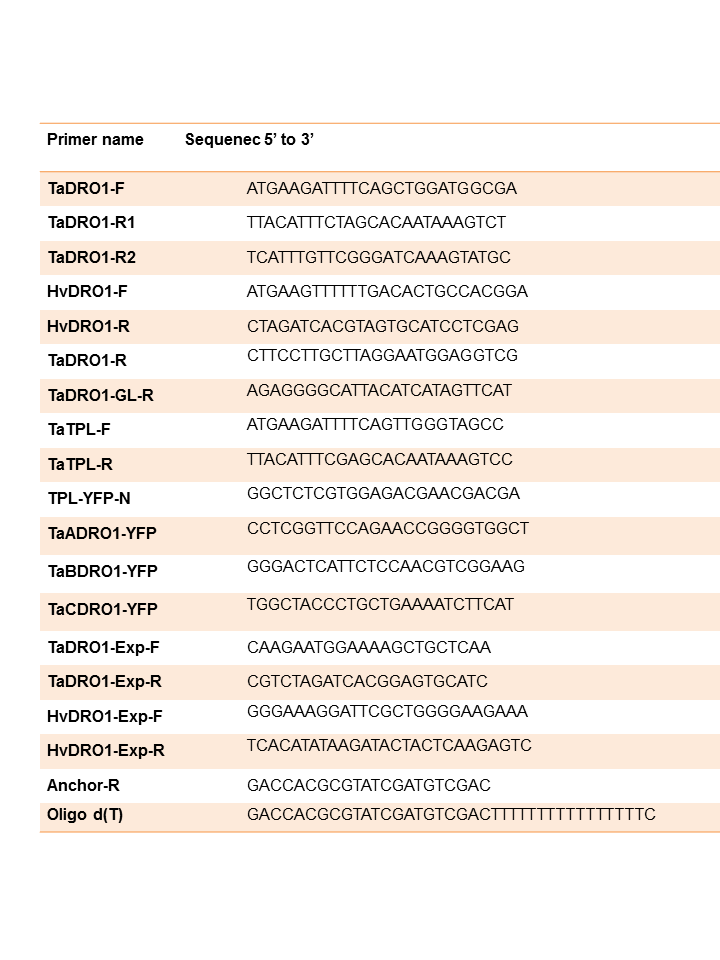

Supplement: S1 Table — Primers used for different experiments including cDNA synthesis, gene isolation, expression and protein-protein interactions are shown. The sequence direction is 5’ to 3’. (TIF) [file pone.0214145.s014.TIF]

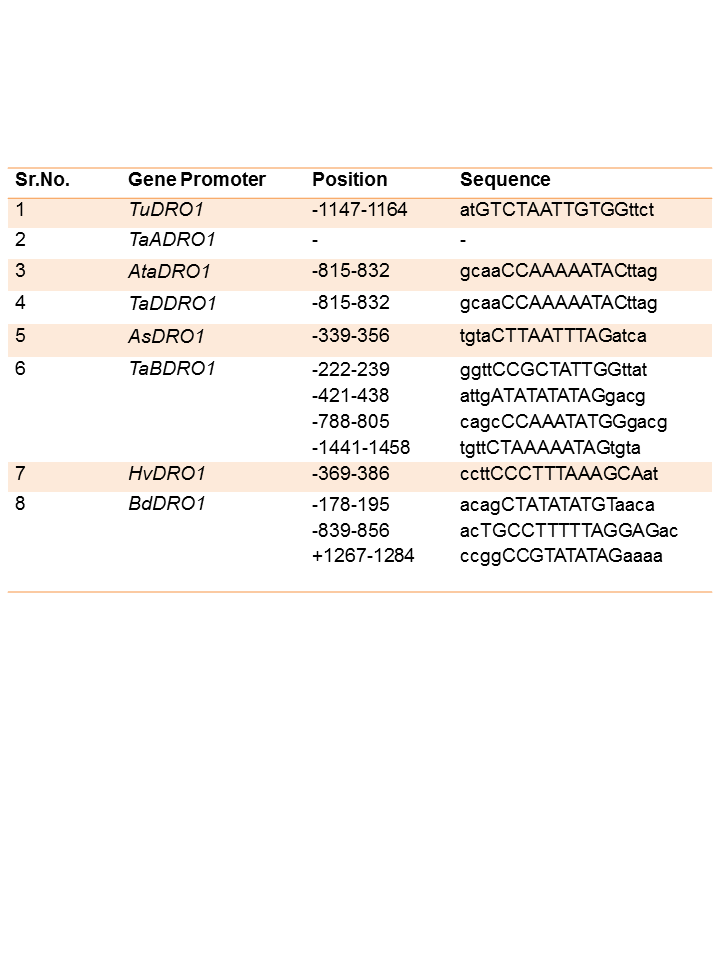

Supplement: S2 Table — Different types of CArG-boxes were detected using Mulan program and their positions were marked. The position of these elements was determined by taking first nucleotide upstream of ATG as -1bp. The core sequence motif is mentioned in capital letters. (TIF) [file pone.0214145.s015.TIF]

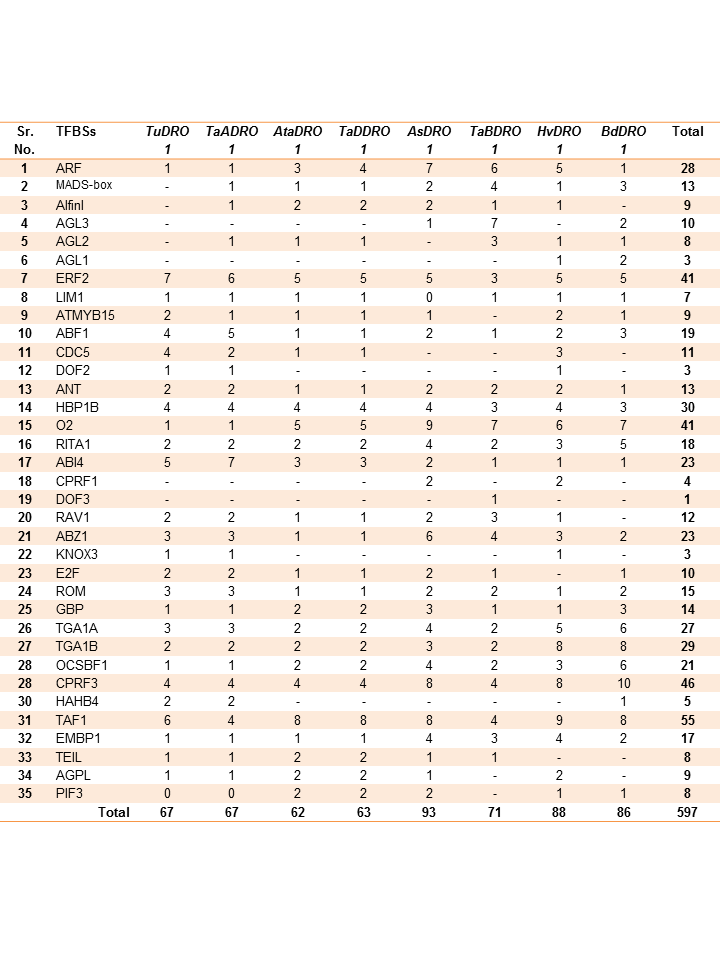

Supplement: S3 Table — For identification of TFBSs, -2kb sequence upstream of translational start site was selected as promoter and analyzed using Mulan program. The position and number of times occurrence of different TFBSs were recorded. The “-” sign indicates the absence of a particular site in that promoter sequence. The total number of TFBSs and their occurrence was also documented as highlighted in bold. (TIF) [file pone.0214145.s016.TIF]
