# Supplementary material for: Evolution of Deeper Rooting 1-like homoeologs in wheat entails the C-terminus mutations as well as gain and loss of auxin response elements
Source: PLoS One. 2019 Apr 4;14(4):e0214145. doi: 10.1371/journal.pone.0214145 (PMC6448822; doi:10.1371/journal.pone.0214145)
Supplement: S1 File — Raw data for measurement of root morphology (Fig 1B and 1C), root angle (Fig 1E) and real time gene expression (Fig 4A–4F) analysis. (ZIP) [file pone.0214145.s017.zip › Supporting Information/Submitted GenBank Accessions.docx]

**GenBank Accession Numbers for Submitted Sequences**

1. TaANDRO1-Like BankIt2203805 Seq       MK639010
2. TaANDRO1-Like BankIt2203831 Seq1     MK639011
3. TaANDRO1-Like BankIt2203854 Seq1    MK639012
